# Screening and Biocontrol Potential of Rhizobacteria Native to Gangetic Plains and Hilly Regions to Induce Systemic Resistance and Promote Plant Growth in Chilli against Bacterial Wilt Disease

**DOI:** 10.3390/plants10102125

**Published:** 2021-10-07

**Authors:** Abhijeet Shankar Kashyap, Nazia Manzar, Mahendra Vikram Singh Rajawat, Amit Kumar Kesharwani, Ravinder Pal Singh, S. C. Dubey, Debasis Pattanayak, Shri Dhar, S. K. Lal, Dinesh Singh

**Affiliations:** 1 Division of Plant Pathology, Indian Agricultural Research Institute, New Delhi 110012, India; amitmicro1@gmail.com (A.K.K.); ravinder.20033@gmail.com (R.P.S.); 2 Plant Pathology Lab, ICAR-National Bureau of Agriculturally Important Microorganisms, Maunath Bhanjan 275103, India; naziamanzar786@gmail.com (N.M.); rajawat.mvs@gmail.com (M.V.S.R.); 3 Division of Plant Quarantine, ICAR-NBPGR, New Delhi 110012, India; scdube2002@yahoo.co.in; 4 Division of Plant Biotechnology, ICAR-NRCPB, New Delhi 110012, India; debasispattanayak@yahoo.co.in; 5 Division of Vegetable Science, Indian Agricultural Research Institute, New Delhi 110012, India; shridhar.iari@gmail.com; 6 Division of Genetics, Indian Agricultural Research Institute, New Delhi 110012, India; skla68@gmail.com

**Keywords:** *Pseudomonas fluorescens* PDS1, biocontrol, induced systemic resistance, chilli bacterial wilt, *Ralstonia solonacearum*, PGPR, defense-related enzymes, *Bacillus subtilis* KA9

## Abstract

Plant growth-promoting rhizobacteria (PGPR) is a microbial population found in the rhizosphere of plants that can stimulate plant development and restrict the growth of plant diseases directly or indirectly. In this study, 90 rhizospheric soil samples from five agro climatic zones of chilli (*Capsicum annuum* L.) were collected and rhizobacteria were isolated, screened and characterized at morphological, biochemical and molecular levels. In total, 38% of rhizobacteria exhibited the antagonistic capacity to suppress *Ralstonia solanacearum* growth and showed PGPR activities such as indole acetic acid production by 67.64% from total screened rhizobacteria isolates, phosphorus solubilization by 79.41%, ammonia by 67.75%, HCN by 58.82% and siderophore by 55.88%. We performed a principal component analysis depicting correlation and significance among plant growth-promoting activities, growth parameters of chilli and rhizobacterial strains. Plant inoculation studies indicated a significant increase in growth parameters and PDS1 strain showed maximum 71.11% biocontrol efficiency against wilt disease. The best five rhizobacterial isolates demonstrating both plant growth-promotion traits and biocontrol potential were characterized and identified as PDS1—*Pseudomonas fluorescens* (MN368159), BDS1—*Bacillus subtilis* (MN395039), UK4—*Bacillus cereus* (MT491099), UK2—*Bacillus amyloliquefaciens* (MT491100) and KA9—*Bacillus subtilis* (MT491101). These rhizobacteria have the potential natural elicitors to be used as biopesticides and biofertilizers to improve crop health while warding off soil-borne pathogens. The chilli cv. Pusa Jwala treated with *Bacillus subtilis* KA9 and *Pseudomonas fluorescens* PDS1 showed enhancement in the defensive enzymes PO, PPO, SOD and PAL activities in chilli leaf and root tissues, which collectively contributed to induced resistance in chilli plants against *Ralstonia solanacearum*. The induction of these defense enzymes was found higher in leave tissues (PO—4.87-fold, PP0—9.30-fold, SOD—9.49-fold and PAL—1.04-fold, respectively) in comparison to roots tissue at 48 h after pathogen inoculation. The findings support the view that plant growth-promoting rhizobacteria boost defense-related enzymes and limit pathogen growth in chilli plants, respectively, hence managing the chilli bacterial wilt.

## 1. Introduction

Chilli (*Capsicum annuum* L.) is the world’s most popular spice and India’s most frequently produced spice [1]. Chilli crop quality and yield are primarily influenced by biotic factors such as aerial-borne and soil-borne plant pathogens. In chilli, bacterial wilt is a significant problem caused by *Ralstonia solanacearum*, a soil-borne pathogen and has made its management difficult.

*R. solanacearum* is widespread in agro-ecological zones of India and causes severe lethality in chilli and other solanaceous crops such as tomatoes, eggplants and potatoes. *R. solanacearum* is presently the most thoroughly investigated phytopathogenic bacterium [2,3,4] and because of its extensive host range, bacterial wilt is challenging to manage and withstand in various environments, such as irrigation, soil and water. It is usual to see host plant resistance breakdowns due to significant genotype–environment interactions. Moreover, various methods, notably chemicals, have been evaluated and shown to be the most flexible and cost-effective plant disease treatment, but no effective chemical solution to address this soil-borne plant pathogen is presently available that makes the bacterial wilt disease an expensive concern for farmers [5].

In a real era of sustainable agriculture, fiber, food and fuel will be required to meet the demands of an ever-increasing global population. It is likely to prioritize a “Fresh” Green Revolution, maybe the Bio-Revolution, on less expensive inputs with decreased environmental effects. Therefore, biological control using rhizobacteria should be considered an alternative approach to managing soil-borne plant pathogens, including *R. solanacearum*. The use of microbes-based agricultural inputs has a long-standing record [6]. *Glomus*, *Pseudomonas*, *Bacillus* and many other strains have been commercially produced in recent years. The need for bacterial taxa *Pseudomonas* [7,8], *Bacillus* [9], *Lactobacillus* [10] and *Actinobacteria* [11] in crop cultivation has been evaluated for plant growth-promoting traits to enhance agricultural production. *Rhizobacteria* such as *Pseudomonads*, *Arthrobacter*, *Bradyrhizobium*, *Bacillus*, *Enterobacter*, *Alcaligenes* and *Serratia* enhanced the plant growth-promoting attributes and acted as a potent antagonistic agent against the plant pathogen [12,13,14,15,16]. PGPR may alter the performance of the plant by direct or indirect methods. Direct mechanisms perform through the production of phytohormones, enhanced availability and utilization of nutrients in soil via chelation of nutrients (Fe) through secretion of siderophore, biological nitrogen fixation, solubilization of fixed form of nutrients to plant-usable form (P, K, Zn) and so on [17,18]. Indirect plant growth-promotion activities are involved in the protection of the plants from the adverse effects of the plant pathogens [19]. There are three main ways in which PGPR works: by synthesizing specific compounds for plants, by making it easier for plants to take nutrients from the soil and by mitigating pathogens of the plant [20,21]. *Pseudomonas fluorescence* is a well-known biocontrol agent that is an effective colonizer when directly inoculated into seeds and soil [22,23]. Several antagonistic mechanisms are produced by rhizobacteria (siderophores, antibiotics, hydrolytic enzymes and volatile compounds) for survival and competition [24,25]. The phytohormones such as indole acetic acid, cytokinins and gibberellins have a synergistic impact on the inhibition of plant pathogens along with plant growth promotion [26,27,28,29,30,31,32]. *Bacillus subtilis* is a widely accepted rhizosphere and soil inhabitant bacteria with double benefits, including promoting plant growth and acting as an antagonistic potential [33,34,35]. *Bacillus cereus* also demonstrated strong biocontrol potential against a wide range of plant pathogens, including *Ralstonia solanacearum*, which causes bacterial wilt in solanaceous crops [36]. Phosphate solubilization is among the first consequences of PGPR for plant nutrition. The P transformation of the soil, including P solubilization necessary for plant growth, involves rhizobacteria [37]. The capacity of phosphate-solubilizing bacteria to solubilize and mineralize P has a holistic approach. *Bacillus*, *Pseudomonas*, *Erwinia*, *Flavobacterium* and *Rhizobium* are able to solubilize phosphates [38]. Iron is also available in vast amounts in the soil, but it is not available for plants such a phosphorous. Several bacterial strains improve the abundance of Fe by producing organic acids or siderophores [39,40,41]. Siderophores are extracellular, low molecular weight compounds with affinity to ferric iron, secreted by microorganisms to take iron from the external environment [42]. They inhibit harmful plant pathogens with iron competition [43]. Siderophores may operate as iron chelators, forming soluble complexes taken up by plants, or they may make them inaccessible to phytopathogenic bacteria by binding to the accessible form of iron in the soil [44]. Indole acetic acid (IAA) is a phytohormone that regulates plant growth and functions as a signal molecule [45,46,47]. Auxin plays an important role for healthy interaction between PGPR and beneficial plant. For example, PGPR strains *Aeromonas punctata* PNS-1, *Azospirillum brasilense* Sp245 and *Aeromonas punctata* PNS-1, which produce auxin, helped to enhanced growth and induce the morphological variations in *A. thaliana* [48,49,50]. Although most physiological parameters in the plant are associated with these plant hormones directly or indirectly, it is not surprising that PGPR can affect the quantity, location and direction of the auxin movement within the plant [51]. Many investigations have discovered that different PGPR strains can generate auxin in culture [51,52]. Additionally, the position of PGPR colonization sites on the root may be a significant consideration. If auxin-producing bacteria effectively colonize the main root or lateral root elongation zone, as well as the zone of lateral root development, the auxin levels in these places may increase in a localized area of the plant [53,54]. Auxin-producing PGPR has also been found to alter the transcription of genes involved in defense, hormone production and cell wall formation [55], increase root biomass, reduce the density and size of stomata [56], activate auxin response genes, enhance longer roots [57] and improve plant growth [58]. The plant gene expression involved in the production, signalling and transport of auxin can be affected by PGPR as well. The IAA synthesis gene expression was increased by the inoculation of *Bacillus* sp. LZR216 and *Phyllobacterium brassicacearum* STM196 PGPR strains [59]. As in the context of biological bacteria VOCs, mixes of volatile compounds that are lacking traditional auxins can be sufficient to promote plant growth through auxin biosynthesis and movement in the plant, without the need for additional auxins [60]. The PGPRs release hormones that enhance root absorption and maintain plant hormonal balance in adverse circumstances, such as drought [61]. Auxins produced by PGPRs improve the root structure and development. A growth-promotion activity, therefore, can be due to changes in plant hormonal pathways, especially the IAA signalling pathway, independently from the capacity of the strain to actually produce IAA [62,63]. A volatile metabolite, hydrocyanic acid (HCN), is considered to have a significant role in the biocontrol of soil-borne diseases [64]. It prevents the transfer of electrons, thus destroying the energy supply to the cells, contributing to the organism’s death. Many researchers have documented HCN production from *P. aeruginosa*, *P. fluorescens* and other rhizobacteria [65]. It was found essential in the biological control of *P. fluorescens* [66]. 

PGPR promotes the innate immunity in plants by enhancing the physical and biochemical responses of the plant against environmental stresses, hence promoting an induced systemic resistance. The production of defense-related enzymes elevated after the interaction of PGPR with the host plant. As a result, an increase in the defensive enzyme activities in the plant provides a greater chance of survival under high stress conditions [67]. For the detection of different microorganisms, microbiology labs require fast methods. The 16S rRNA gene sequences are used as a regular marker to identify bacteria and their phylogenetic relationship. This is found in all prokaryotic cells and it has retained variable sequence regions, essential for the simultaneous universal amplification and measurement of both near and distant phylogenetic relationships, which evolve at very different rates. These characteristics allow 16S rRNA to assign close relationships to the genus and, in some cases, to identify species [68]. For accurate information, the 16S rRNA gene (1500 bp) is sufficient [69].

Information about the indigenous bacterial population, their identification and characterization are necessary to know the abundance and distribution of bacterial communities in specific crop rhizospheres [70,71]. As pesticides are used in agricultural practices, it is critical to look for local microbial strains that can be used as growth-friendly inoculums to maximize crop production [72]. Recently, bacterial diversity has been investigated and recorded in north-eastern India [73], yet minimal data are available on the rhizosphere microbiome of chilli native to hilly regions of north-western India and the Gangetic Plains. Therefore, in recent years, beneficial microorganisms such as PGPR as biofertilizers and biocontrol agents have become more important to improve plant growth and manage plant diseases with an eco-friendly approach. In order to focus on the said aspects, the present work aims at isolating and characterizing the rhizospheric bacteria having antagonistic potential against *R. solanacearum* and the determination of plant growth-promoting attributes.

## 2. Results

### 2.1. Isolation and Diversity Index of Antagonistic Rhizobacteria

**Figure 1:**
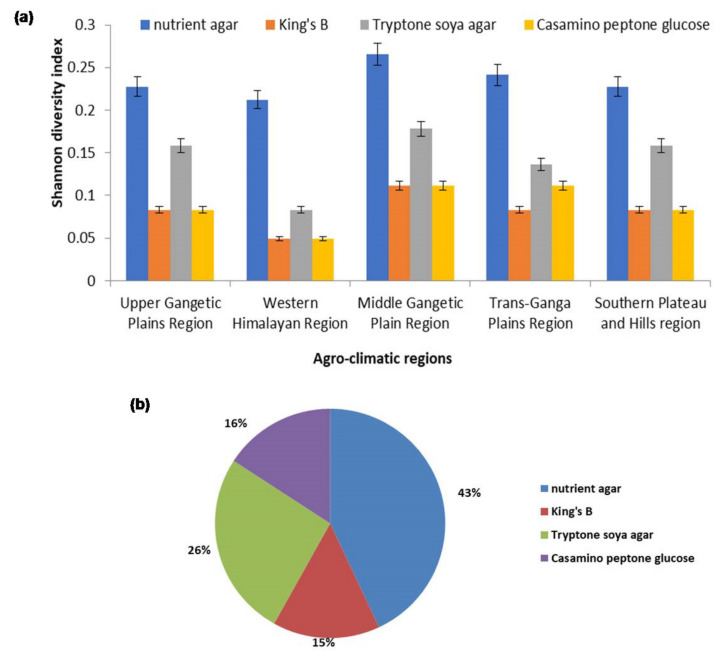
(**a**) Diversity index of rhizobacterial population isolated from rhizospheric soil samples from different agroclimatic zones on different microbiological media. The results are expressed as average of three replications and error bars indicate standard deviation of the means; (**b**) Shannon diversity index to access the abundance of rhizobacterial population on different media.

Chilli rhizospheric soil was collected from five agro-climate regions: the Western Himalayan Region, the Southern Plateau and Hills Region, the Upper Gangetic Plains Region, the Middle Gangetic Plain Region and the Trans-Gangetic Plains Region, with a wide latitude (13.63° N–9.57° N) and longitude range (74.98° E–84.12° E) and the geographical coordinates, location, country and other details are given in Table 1. Rhizobacteria were isolated on four different types of media, King’s B (KB), Tryptone soya agar (TSA), Nutrient agar (NA) and Casamino peptone glucose (CPG) medium and several bacterial colonies were observed in these media. The abundance of the rhizobacterial population was calculated using Shannon diversity index in the chilli rhizospheric soil samples. In total, 63 rhizobacterial isolates were identified and characterized from various chilli rhizospheric soil samples. In total, 17 isolates from Karnataka, 14 isolates from Andhra Pradesh, 11 from Uttarakhand, 9 from Uttar Pradesh, 7 from Bihar and 5 from Delhi were selected and screened for various morphological characteristics such as texture, size, margin, shape and pigmentation, as described in Supplementary Materials Table S1. The microorganisms found had a smooth-rough-slim texture and a color spectrum ranging from pure white to transparent light brown to red-cream-yellow. The chilli rhizospheric microbial community harvested at full strength from the Middle Gangetic Plain Region had the highest microbial diversity in comparison with other geographical locations (measuring the heterotrophic counts of microbes on NA agar plates, with microbial richness (12 ± 1) and Shannon diversity index (0.27 ± 0.04)) and were selected for the microbial diversity research. The overall maximum diversity (43%) was observed in NA with Shannon diversity index (1.17 ± 0.27), followed by TSA (0.71 ± 0.52), CPG (0.43 ± 0.39) and KB (0.41 ± 0.24), respectively (Figure 1a,b; Supplementary Table S2). 

### 2.2. In Vitro Antagonistic Activity of Rhizobacteria against Chilli Wilt Causing Pathogen Ralstonia solanacearum

**Figure 2:**
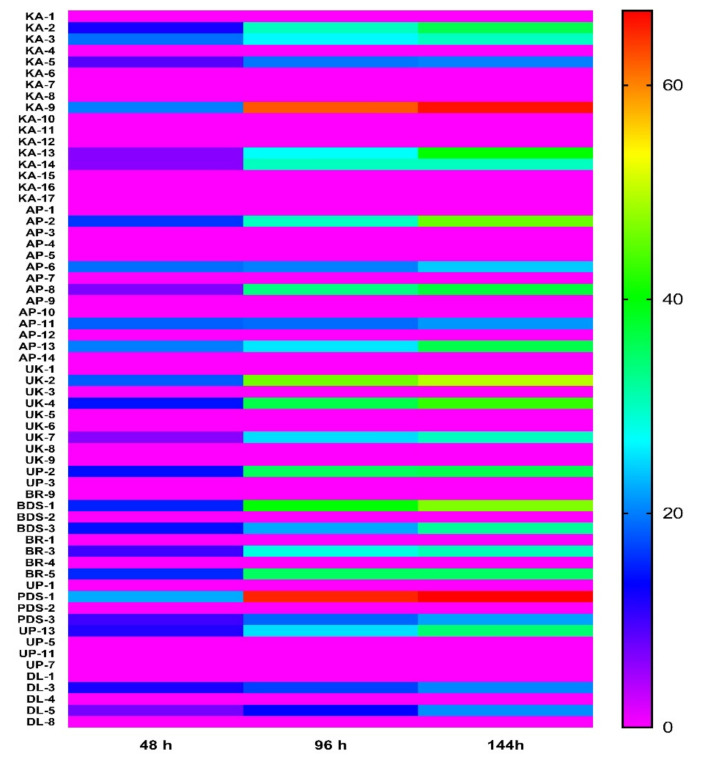
Biocontrol efficacy of fungal strains isolated from different regions at different time intervals (48 h, 96 h and 144 h). The results are expressed as average of three replications.

Out of the 63 isolates of rhizobacteria isolated from rhizospheric soil of chilli plants, 38.09% rhizobacteria were found to have antagonistic activity against *R. solanacearum*. A marked inhibition in the growth of *R. solanacearum* culture was observed in the presence of rhizobacterial isolates. The maximum inhibition zone (22.4 mm diameter) of *R. solanacearum* was found by the isolate PDS1 isolated from the Middle Gangetic Plain region, followed by KA9 isolate (20.06 mm diameter). However, a minimum inhibitory effect (6.12 mm diameter) was exhibited by UK-7 of Uttarakhand after 48 h. The majority of the rhizobacterial isolates (39) did not inhibit the growth of *R. solanacearum* (Figure 2).

### 2.3. Morphological and Biochemical Characterization of Rhizobacteria

The most potent antagonist was selected, 24 isolates (KA2, KA3, KA5, KA9, KA13, KA14, AP2, AP6, AP8, AP11, AP13, UK2, UK4, UK7, UP2, BDS1, BDS3, BR3, BR5, PDS1, PDS3, UP13, DL3 and DL5), along with 10 non-antagonist isolates (KA7, KA15, KA17, UK6, UK8, UP3, BR4, UP7, DL1 and DL8), which were characterized based on colony features, as well as morphological, biochemical and molecular ones. Morphological traits and biochemical tests were conducted on all of the isolates. Size, shape, margin, opacity, elevation, texture and pigmentation are all morphological traits. All of the rhizobacteria identified have a variable size, large to small, having different shapes, viz., round, irregular, uniform with transparent, opaque to semi-transparent opacity and smooth-rough texture with the broad color spectrum of pure white-translucent, brown-greenish-yellow and grey-white. The colonies vary in size from large to small, having different shapes, viz., round, irregular, uniform with transparent, opaque and semi-transparent opacity.

**Figure 3:**
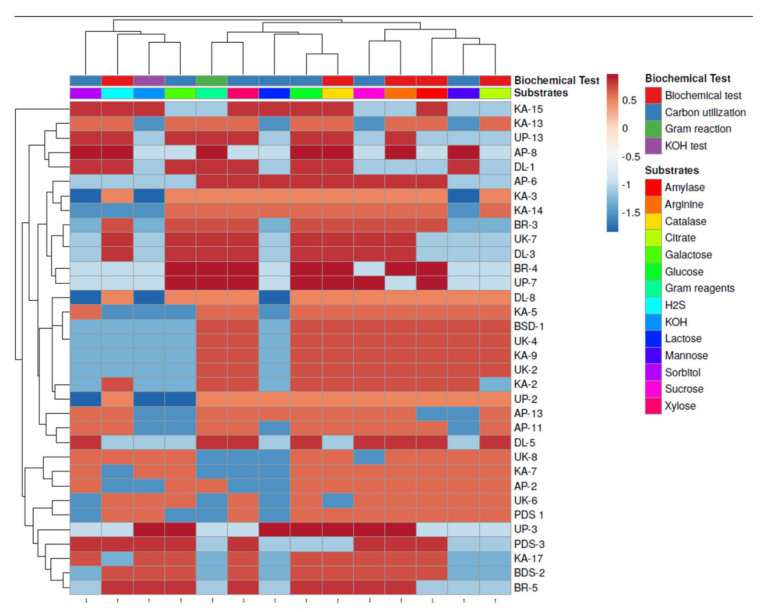
Clustering analysis and heatmap based on biochemical characterization of rhizobacterial strains isolated from different regions. The results are expressed as average of three replications.

**Figure 4:**
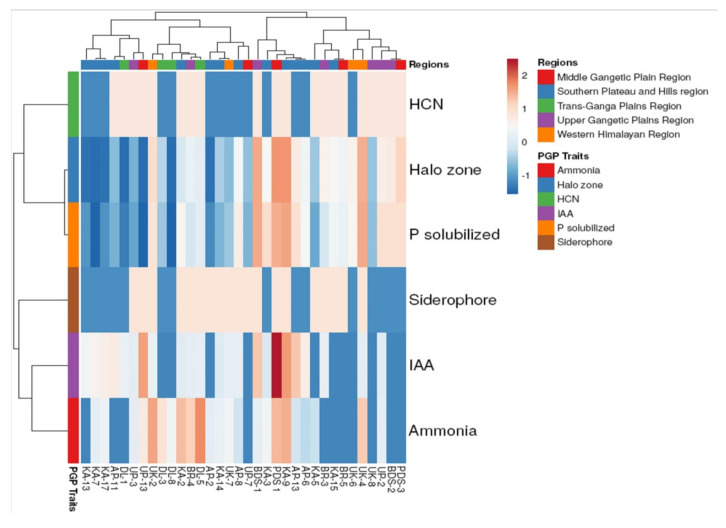
Clustering analysis and heatmap based on plant growth-promoting activities of rhizobacterial strains isolated from different regions.

Biochemical tests, including gelatine liquefication, starch hydrolysis, H_2_S, arginine, citrate, KOH, Oxidase and catalase, as well as plant growth promotion (PGP) characteristics, such as indole acetic acid (IAA), siderophores, ammonia synthesis, HCN and phosphate solubilization, were screened for isolates; details are given in Figure 3 and Figure 4. Out of the 34 isolates of rhizobacteria, 29.42% of rhizobacteria were found to be gram-negative and 70.58% of bacteria, were gram positive.

**Figure 5:**
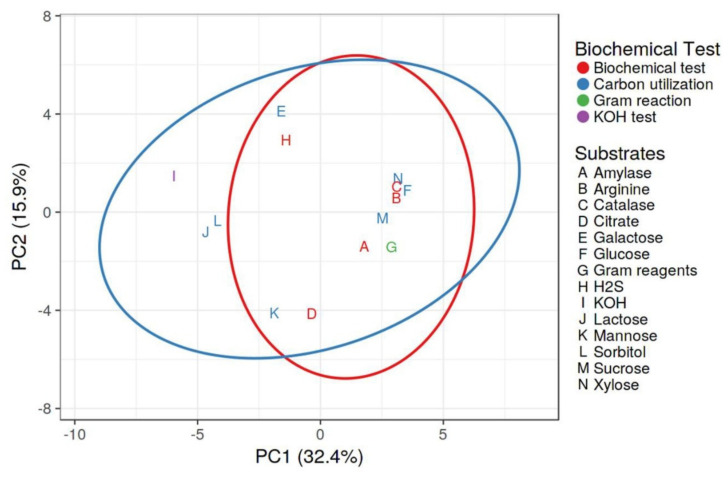
Principal component analysis depicting correlation and significance between biochemical tests and substrate utilization profiling of rhizobacterial strains.

The majority of the isolates (24 isolates) were rod-shaped, whereas 10 isolates were coccus-shaped. The use of glucose as a carbon source was found in 29.42% of rhizobacteria. Galactose was utilized as a carbon source by 58.88% of the isolates. Rhizobacteria used xylose and sucrose in proportions of 82.35% and 76.47%, respectively. In biochemical tests, 91.17% of the isolates tested positive for catalase, while 76.47% tested positive for amylase. Citrate utilization and H_2_S generation were measured, with 52.59 and 58.82% of rhizobacteria isolates demonstrating favorable results in citrate utilization and H_2_S production. Arginine dehydrogenase synthesis was detected in 91.17% of rhizobacteria. In Figure 3, it is revealed that none of the rhizobacteria utilized all the carbon sources provided to them as glucose, galactose, sorbitol, mannose, xylose and sucrose. It suggests that a diversity of bacteria live in the rhizosphere of the chilli plants and that they behave differently in terms of carbon sources and enzyme production. Principal component analysis was performed (Figure 5) through Clustvis 2.0 online program. Two principal components PC1 and PC2 were observed with the variance level of 32.4% and 15.9%, respectively. Variables of biochemical tests and different substrates among 34 rhizobacterial strains were highly correlated with PC1 and PC2 and residing into the same eclipse (excluding KOH, lactose and sorbitol) at 95% significance level. Besides, variables KOH, lactose and sorbitol were more correlated with carbon utilization profile. The results concluded that rhizobacterial strains comprising eclipse I and eclipse II showed significant positive responses to various biochemical characterization.

### 2.4. PGPR Characteristics of Rhizospheric Bacteria

In this study, indole acetic acid was produced by 67.64% of 34 rhizobacteria isolates, phosphorus solubilization by 79.41%, ammonia by 67.75%, HCN by 58.82% and siderophore by 55.88%. In vitro, isolates AP13, BDS1, UP13 and PDS1 were strong producers of IAA, whereas isolates AP13, BDS1, UP13 and PDS1 were medium producers of IAA. Quantitative estimation determines the maximum IAA-producing isolates, were PDS1 (7.26 ± 0.32 µg mL^−1^) and KA9 (5.65 ± 0.19 µg mL^−1^) and the minimum was observed in KA5, KA15, AP2, UK6, UK8, BDS2, BR5, PDS3, UP7, DL3 and DL8. More than 75% of rhizobacteria isolates solubilized phosphorus, a crucial nutritional element for plants. However, rhizobacteria dominated this action; 6 of the 34 isolates in this research were able to solubilize phosphate by forming a 10 mm clear halo zone surrounding the colonies. For the quantitative measurement of phosphate solubilization, PGPR strains were selected based on their solubilizing zone (SZ). Isolates including KA9 (13.40 ± 0.61 mm and 5.92 ± 0.07 mg mL^−1^) and PDS1 (13.39 ± 0.50 mm and 5.77 ± 0.28 mg mL^−1^) showed maximum SZ and solubilized phosphorus, respectively, while DL1 (0.01 ± 0.01 mm and 0.52 ± 0.18 mg mL^−1^) showed minimum SZ and solubilized phosphorus, although some isolates showed no phosphorus production (KA7, DL8 and UP13), as described in Table 2. About 58% of isolates of rhizobacteria produced hydrogen cyanide and all isolates showing positive in HCN production are weak in production of HCN. Moreover, 55.12% of rhizobacteria isolates produced siderophores, with one isolate, PDS1, being a high producer of siderophores, which may contribute to iron chelation and the production of soluble complexes taken up by plants. Ammonia production is one of the activities of rhizobacteria and 6 isolates, KA9, UK2, UK4, BR4, PDS1 and DL5, were shown to be medium producers of ammonia in a qualitative analysis of 34 isolates. Later quantitative determination of ammonia production was estimated and found maximum by isolates such as UK2 (4.98 ± 0.08 mol mL^−1^) and KA9 (4.80 ± 0.09 mol mL^−1^). Many isolates were unable to produce ammonia, viz., KA13, UK6, AP11, UK8, BDS2, BR3, BR5, PDS3, UP7 and DL1. Only 26.47% of the 34 isolates, namely KA2, KA9, UK2, UK4, BDS1, BDS2, BR4, PDS1 and DL5, were able to generate all plant growth-promoting characteristics such as siderophores, indole acetic acid, ammonia, hydrogen cyanide and phosphate solubilization under in vitro condition. The findings show that, while not all rhizobacteria have the ability to stimulate plant development, specific isolates have the potential to exhibit such characteristics. Figure 4 depicts that, based on different regions, data were grouped into two clusters: cluster I and cluster II. Cluster I was mainly comprised only four regions (excluding Trans-Ganga Plains Region) and cluster II comprised selected regions. Both cluster I and II were further grouped into two sub-clusters individually. Overall, cluster I represents the rhizobacterial strains having a high efficiency of plant growth-promoting activities compared with cluster II. While clustering based on plant growth-promoting activities, data were grouped into two clusters. However, statistical analysis showed that strains PDS1, KA9, BDS1 and UK4 were the most promising candidates for plant growth-promoting activities obtained from different regions. Rhizobacterial strain PDS1 showed the highest production of IAA, followed by strain KA9, BDS1 and AP-13. Besides, fungal strains PDS1, KA9, BDS1 and UK4 were showed to have the utmost similar degree of phosphorus released from insoluble tri-calcium phosphate. However, the HCN production and halo zone formation on Pikovaskaya agar plate by rhizobacterial strain were less than other plant growth-promoting activities.

### 2.5. Biocontrol of Bacterial Wilt Disease and Plant Growth-Promotion Attributes

**Figure 6:**
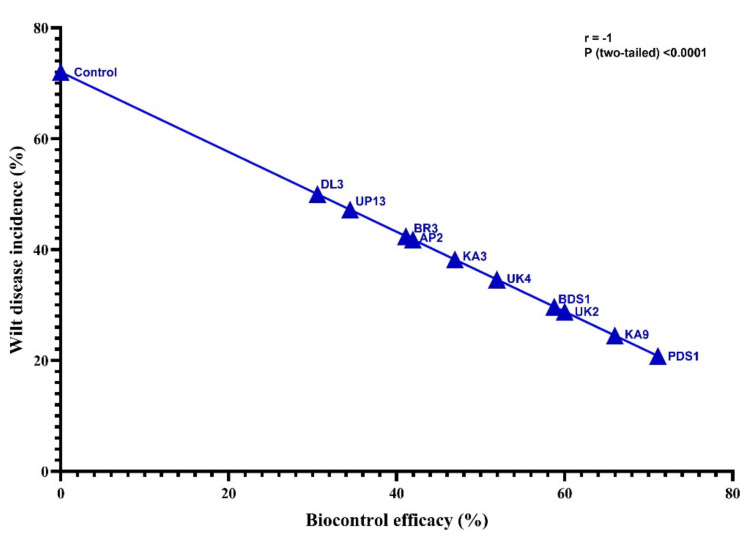
Correlation between wilt disease incidence and biocontrol efficacy of rhizobacterial strains on chilli crops.

The bacterial communities in the rhizospheric soil of the chilli plant were hostile to *R. solanacearum* and promoted plant development under in vitro conditions. The 10 most promising rhizobacteria used in this investigation were BDS1, KA3, KA9, UK2, BR3, UP13, AP2, PDS 1, UK4 and DL3 based on their antagonistic and plant growth-boosting capacities. Under glasshouse conditions at the National Phytotron Facility, IARI, New Delhi, these isolates of rhizobacteria were evaluated for their bio-efficacy against bacterial wilt disease induced by *R. solanacearum* and plant growth boosting activities in chilli cv. Pusa Jwala (susceptible cv.). The minimum bacterial wilt incidence was 20.8%, with the highest biocontrol efficacy of 71.11% recorded in chilli cv. Pusa Jwala treated by PDS 1 followed by KA9 (24.5%), UK2 (28.8%), BDS1 (29.7%), UK4 (34.6%), KA3 (38.2%), BR3 (41.8%), AP2 (42.4%), UP13 (47.2%) and DL3 (50.0%) with the isolates biocontrol efficacy of KA9 (65.97%), UK-2 (60.00%), BDS 1 (58.75%), UK 4 (51.93%), KA3 (46.94%), BR3 (41.94%), AP2 (41.10%), UP13 (34.44%) and DL3 (30.56%) of chilli rhizobacteria (Table 3). After 10 days of inoculation, the bacterial wilt disease signs appeared in *R. solanacearum* UTT25 infected plants, but the plant treated with the rhizobacteria delayed the emergence of wilt disease by 10–12 days. Furthermore, there was a substantial difference in the decrease in wilt disease in chilli under glasshouse settings among these isolates (*p* > 0.05). All rhizobacterial strains showed significant biocontrol efficacy as compared to control. These rhizobacteria isolates extend the potential to reduce the wilt disease incidences that ranged from 30% to 75%. Among the rhizobacterial strains, strain PDS1 showed the highest potential for reducing wilt disease incidences, followed by KA9, UK2, BDS1, UK4 and KA3 strains. The DL3 strain showed the least potency in comparison to other strains. Statistical analysis revealed that wilt disease incidence and biocontrol efficacy of selected fungal strains were inversely correlated (r = −1) with each other at *p* < 0.0001 level (Figure 6).

Rhizobacteria have an impact on plant development by producing a number of plant growth-promoting compounds. After 40 days of treatment, plant growth-boosting parameters such as plant length, dry weight of root and shoot and growth-promoting efficacy were measured. In plant growth-promotion abilities, maximum shoot length 19.7 cm was recorded in PDS1 followed by KA9 (18.8 cm), UK2 (16.1 cm), BDS1 (15.6 cm), AP2 (15.6 cm), BR3 (14.8 cm), UP-13 (14.5 cm), KA3 (14.2 cm), UK 4 (13.2 cm) and DL3 (13.4 cm) and minimum shoot length was recorded in treatment of control (12.3 cm). Growth-promotion efficiency was evaluated based on shoot length and maximum GPE (60.16%) was recorded in PDS1 followed by KA9 (52.84%), UK2 (30.89%), BDS1 (26.82%), AP2 (26.82%), BR3 (20.32%), UP13 (17.88%), KA3 (15.44%), DL3 (8.94%) and UK 4 (7.31%). Maximum root length was recorded in isolate PDS1 (12.20 cm) treated plants followed by KA9 (11.46 cm), UK2 (9.82 cm), BDS 1 (9.61 cm), AP2 (9.42 cm), UP13 (8.21 cm), KA3 (8.16 cm), BR3 (8.19 cm), DL3 (7.30 cm) and UK4 (7.18 cm) and minimum root length was recorded in control treatment (7.02 cm) after 40 days of inoculation. 

**Figure 7:**
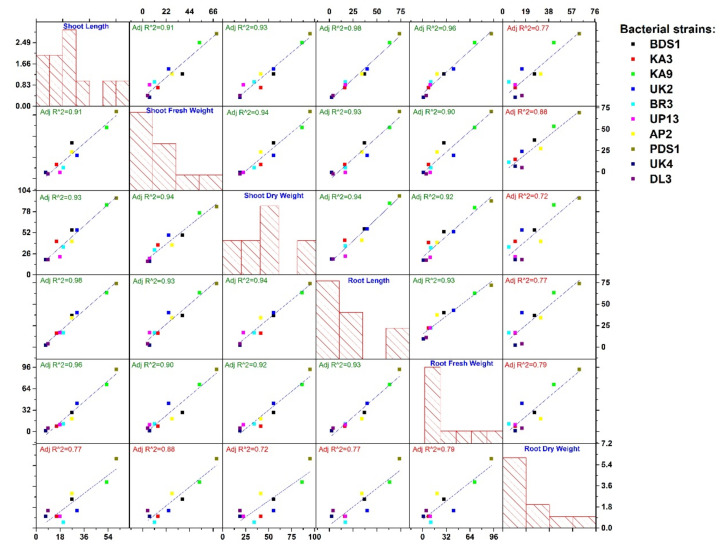
Growth-promotion efficiency of rhizobacteria strains on various growth parameters of chilli and regression analysis.

Isolate PDS1-treated plants had the highest root dry weight (0.31 g), followed by KA9 (0.27 g), AP-2 (0.25 g), BDS 1 (0.24 g), UK2 (0.22 g), DL3 (0.22 g), UP13 (0.21 g), KA3 (0.21 g), UK4 (0.21 g), BR3 (0.20 g) and control plants (0.19 g). The growth-promoting efficacy for root dry weight was noticeably higher (63.15%) in isolate PDS1-treated plants followed by KA9 (42.10%), AP2 (31.57%), BDS1 (26.31%), DL3 (15.78%), UK2 (15.78%), UP13 (10.52%), KA (10.52%), UK 4 (10.52%) and BR3 (5.26%). Maximum shoot dry weight (1.13 g) was recorded in isolate PDS1 treated plants followed by KA9 (1.08 g), BDS 1 (0.90 g), UK2 (0.90 g), KA3 (0.82 g), AP2 (0.82 g), BR3 (0.78 g), UP13 (0.71 g), UK4 (0.69 g), DL3 (0.69 g) and control (0.58 g). The growth-promoting efficacy for shoot dry weight was noticeable higher (94.82%) in isolate PDS1-treated plants followed by KA9 (86.20%), BDS1 (55.17%), UK2 (55.17%), KA3 (41.37%), AP2 (41.37), BR3 (34.48), UP13 (22.41%), UK4 (18.96) and DL3 (18.96). The overall growth-promotion efficiency among various growth parameters (except root dry weight) by selected rhizobacterial strains was showed to have a high regression coefficient (R^2^ < 0.90). Strain PDS1 showed high potential to improve all selected growth parameters followed by strain KA9. This is while other strains showed different degrees of growth promotion among selected growth parameters. Strain UK4 and DL3 showed the lowest response for growth promotion. Selected strains also showed significant growth promotion of root dry weight, but showed less regression coefficient (R^2^ > 0.90), as represented in Figure 7.

**Figure 8:**
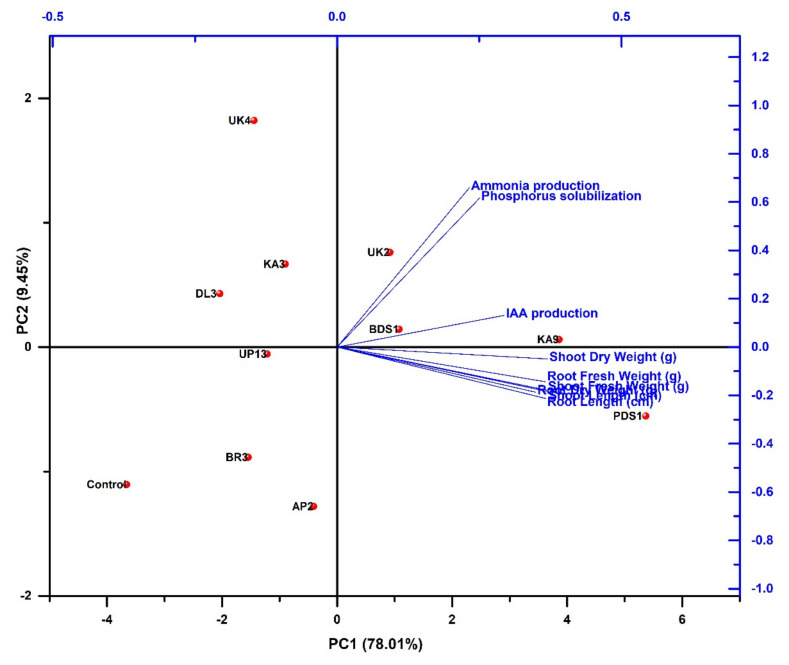
Principal component analysis depicting correlation and significance among plant growth promoting activities, growth parameters of chilli and rhizobacterial strains.

We identified five rhizobacterial isolates (KA9, PDS1, UK2, UK4 and BDS1) that showed the most promising results in terms of biocontrol potential and plant growth promotion based on morphological, biochemical, PGP characteristics and antagonism screening. Principal component analysis (PCA) was performed by using different variables such as plant growth-promoting activities (ammonia production, phosphorus solubilization and IAA production), growth parameters (shoot length, shoot fresh weight, shoot dry weight, root length, root fresh weight and root dry weight) and selected rhizobacterial strains (PD1, BDS1, KA9, UK2, UK4, KA3, DL3, UP13, BR3, AP2 and control). Principal component analysis showed that there are two major principal components (PC1 and PC2). The level of variance of both principal components (PC1 and PC2) were 78.01% and 9.45%. In PCA analysis, rhizobacterial strains were grouped into four clusters: cluster I (BDS1, KA9 and UK2), cluster II (PDS1), cluster III (UP13, BR3, AP2 and control) and cluster IV (UK4, DL3 and KA3). Cluster I was positively correlated with PC1 and PC2, while cluster II was positively correlated only with PC1. Besides, cluster III was negatively correlated with PC1 and PC2, while cluster IV was positively correlated only with PC2. Moreover, cluster II and cluster IV negatively correlated with PC2 and PC1. Cluster I and cluster II showed high correlation with the variables of plant growth-promoting activities and growth parameters, respectively. Cluster III and cluster IV were negatively/less correlated with plant growth-promoting activities and growth parameters, respectively. The results concluded that rhizobacterial strains comprising cluster I and cluster II showed significant improvement in plant growth-promoting activities and growth parameters compared with other strains comprising cluster III and cluster IV (Figure 8).

### 2.6. Molecular-Based Identification of Rhizobacterial Strains 

Molecular characterization was carried out by using amplification of 16S rRNA gene fragments and identified the study strains as PDS1—*Pseudomonas fluorescens* (MN368159), BDS1—*Bacillus subtilis* (MN395039), UK4—*Bacillus cereus* (MT491099), UK2—*Bacillus amyloliquefaciens* (MT491100) and KA9—*Bacillus subtilis* (MT491101). The sequences matched *Pseudomonas* and *Bacillus* spp. sequences in NCBI GenBank with 99–100% similarity. Based on the sequence analysis of the 16S rRNA gene, our identified isolates *Pseudomonas fluorescens* PDS1 has 100% identity with KM589027 (Pf6 strain), *Bacillus subtilis* BDS1 has 100% identity with KF601955 (SBT-014 strain), *Bacillus subtilis* KA9 has 100% identity with MT111081 (MK736123 strain), *Bacillus amyloliquefaciens* UK2 has 100% identity with EF423606 (BCRC14710) and *Bacillus cereus* UK4 has 100% identity with MN148885 (AR2019-1 strain). 

**Figure 9:**
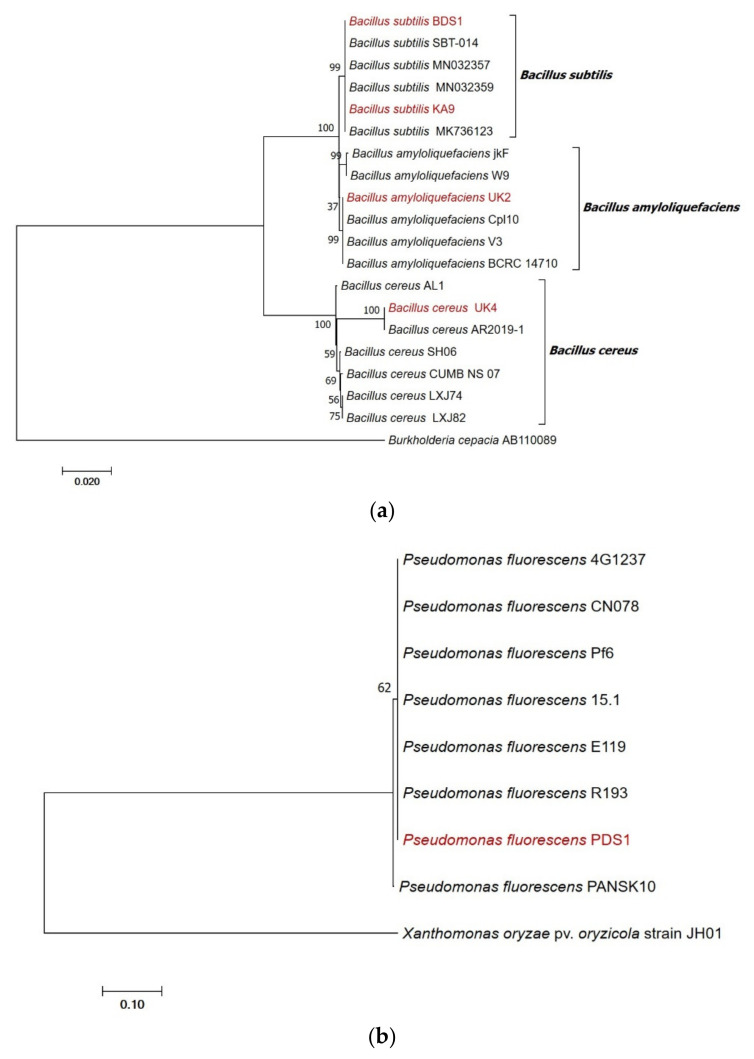
(**a**) Phylogenetic tree highlighting the position of *Bacillus subtilis* strain *BDS1*, *Bacillus cereus* strain UK4, *Bacillus subtilis* strain KA9 and *Bacillus amyloliquefaciens* strain UK2 relative to other type strains within the *Bacillus* genus. Sequences were aligned using Clustal-W and phylogenetic inferences were obtained using the maximum-likelihood method within MEGA 6. Numbers at nodes are percentages of bootstrap values obtained by repeating analysis 1000 times to generate a majority consensus tree. *Burkholderia cepacia* strain *AB110089* was used as an outgroup. (**b**) Phylogenetic tree highlighting the position of *Pseudomonas fluorescens* strain PDS1 relative to other type strains within *Pseudomonas* genus. Sequences were aligned using Clustal-W and phylogenetic inferences were obtained using the maximum-likelihood method within MEGA 6. The numbers at nodes are percentages of bootstrap values obtained by repeating analysis 1000 times to generate a majority consensus tree. *Xanthomonas oryzae* pv. *Oryzicola* strain JH01 was used as outgroup.

The cladogram was constructed and the numerical value presented in the node indicates the bootstrap value. Based on the bootstrap values, the *Bacillus subtilis* (BDS1 and KA9), *Bacillus cereus* (UK4) and *Bacillus amyloliquefaciens* (UK2) isolates with other similar sequences of GenBank could be divided into two separate clades based on 16S rRNA gene sequences. The *Bacillus subtilis* (BDS1) isolate clustered with *Bacillus subtilis* strain-SBT-104, with a bootstrap value of 100%; the *Bacillus subtilis* (KA9) isolate clustered with a *Bacillus subtilis* MK736123 strain with a bootstrap value of 96% and the MN032357, MN032359 strain with a bootstrap value of 66% and 63%, respectively. *Bacillus amyloliquefaciens* (UK2) were clustered with BCRC14710, Cpl 10 and V3, with a bootstrap value of 86%, respectively. *Bacillus cereus* (UK4) is clustered with AR2019-1 with a bootstrap value of 100%. *Burkholderia cepacia* was used as an outgroup (Figure 9a).

Based on the bootstrap values, the *Pseudomonas fluorescens* PDS1 isolate with other similar *Pseudomonas fluorescens* isolates’ sequence of GenBank could be divided into two separate clades based on 16S rRNA gene sequences. The *Pseudomonas fluorescens* PDS1 isolate clustered with *Pseudomonas fluorescens* strains-4G1237, CN078 with a bootstrap value of 95%, R193 with a bootstrap value of 64% and 15.7%, Pf6 with a bootstrap value of 63% and PANSK10 were clustered as distinct clade with a bootstrap value of 95%, respectively. *Xanthomonas oryzae* pv. *oryzicola* was used as an outgroup (Figure 9b).

### 2.7. Overview of Best Five Rhizobacterial Isolates Having Plant Growth Promoting (PGPs) Traits 

The overall characteristic of potential rhizobacteria was summarized in Table 4, isolates PDS1—*Pseudomonas fluorescens* (MN368159), BDS1—*Bacillus subtilis* (MN395039), UK4—*Bacillus cereus* (MT491099), UK2—*Bacillus amyloliquefaciens* (MT491100) and KA9—*Bacillus subtilis* (MT491101) were found to be the most effective PGPR that solubilized insoluble phosphorus and produced IAA, ammonia, HCN and catalase.

### 2.8. The Effect of Rhizobacterial Strains on the Induction of Resistance in Chilli Plant against Bacterial Wilt under Glasshouse Conditions

The chilli cv. Pusa Jwala seeds were bioprimed with *Bacillus subtilis* KA9 and *Pseudomonas fluorescens* PDS1 to study the performance of rhizobacteria for the enhancement of defense-related enzymes such as superoxide dismutase, polyphenol oxidase, phenylalanine ammonium lyase and peroxidase activities against challenged bacterial wilt plant pathogen *R. solanacearum*. These enzymes were estimated at 0, 24, 48, 72 and 96 h after pathogen inoculation (h) in 30-day-old chilli plants.

#### 2.8.1. Peroxidase Activity (PO)

(i) Leaves

**Figure 10:**
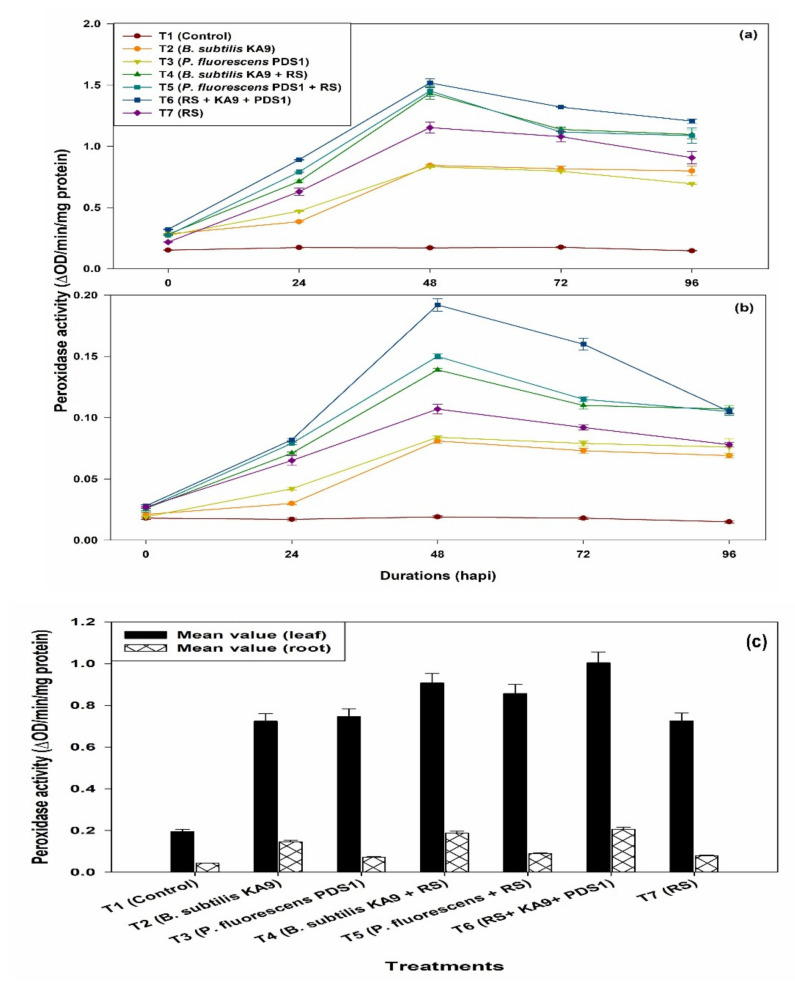
(**a**) The effect of biocontrol agents on the induction of Peroxidase activities in chilli leaves against *R. solanacearum* in glasshouse conditions; observations were recorded after 30 days of sowing. (**b**) The effect of biocontrol agents on the induction of peroxidase activities in chilli roots against *R. solanacearum* in glasshouse conditions; observations were recorded after 30 days of sowing. The results are expressed as average of three replications and error bars indicate standard deviation of the means. (**c**) Mean value comparison of the induction of Peroxidase (PO) activity in between chilli leaves and root against *Ralstonia solanacearum* in glasshouse condition after 30 days of sowing. The results are expressed as average of three replications and error bars indicate standard deviation of the means.

Peroxidase activity was expressed in unit changes in absorbance per minute per milligram protein (ΔOD min^−1^ mg^−1^ protein) in leaves of chilli cv. Pusa Jwala and the maximum PO activity was observed in treatment T6 (1.372 ΔOD min^−1^ mg^−1^ protein) followed by T4 (1.224 ΔOD min^−1^ mg^−1^ protein) at 48 h. The PO activity was increased in all of the treatments at 48 h of pathogen inoculation, after which PO activity declined significantly (Figure 10a). It was noted that only rhizobacteria-treated plants showed more PO activity than untreated plants. Moreover, only *R. solanacearum*-treated plants also showed enhancement of PO activity after 48 h, i.e., 1.050 ΔOD min^−1^ mg^−1^ protein, which was significantly higher than in the untreated plants. However, *B. subtilis* KA9 induced higher PO activity than the *P. fluorescens* PDS1.

(ii) Roots

Overall, maximum PO activity in chilli root was observed in treatment T6 (0.139 ΔOD min^−1^ mg^−1^ protein) followed by T4 (124 ΔOD min^−1^ mg^−1^ protein) at 48 h. The PO activity in chilli roots was increased in all the treatments at 48 h of pathogen inoculation, after which PO activity was declined significantly (Figure 10b). It was noted that only rhizobacteria-treated plants showed more PO activity than untreated plants. Moreover, only *R. solanacearum*-treated plants also showed enhancement of PO activity after 48 h, i.e., 0.116 ΔOD min^−1^ mg^−1^ protein, which was significantly higher than in the untreated plants. However, *B. subtilis* KA9 induced higher PO activity than *P. fluorescens* PDS1. Mean value comparison between the different treatments of the induction of Peroxidase (PO) activities in between chilli leaves and root against *R. solanacearum* in glasshouse condition revealed that treatment T6 showed a maximum induction of PO enzyme in leaves with 1.004 and in root with the mean value of 0.206, respectively; however, the induction of this defense enzyme was found to be much higher in leave (4.87-fold) as compared with roots (Figure 10c)

#### 2.8.2. Polyphenol Oxidase Activity (PPO)

(i) Leaves

**Figure 11:**
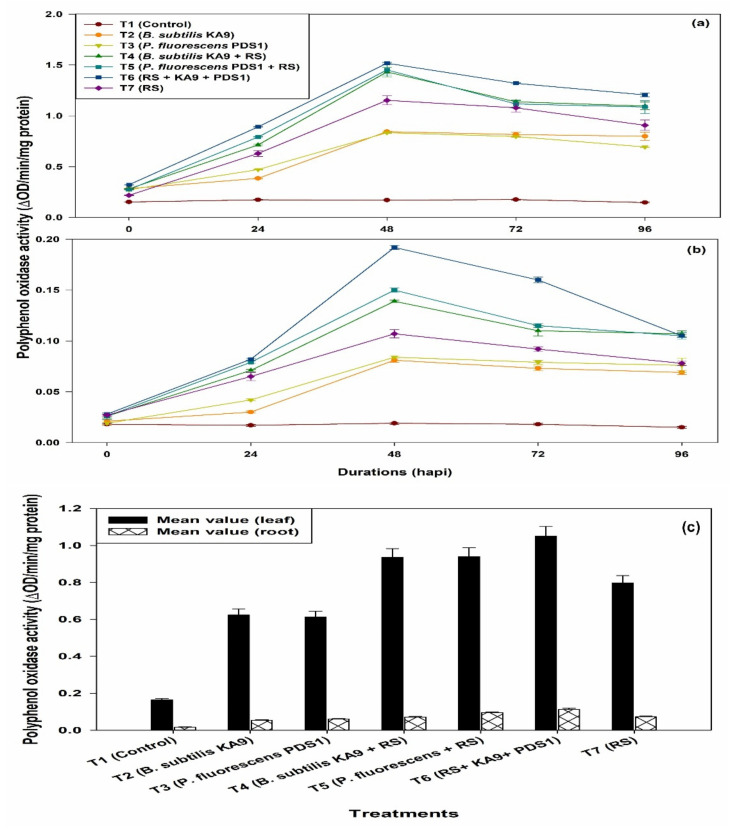
(**a**) The effect of biocontrol agents on the induction of polyphenyl oxidase activities in chilli leaves against *R. solanacearum* in glasshouse conditions; observations were recorded after 30 days of sowing. (**b**) The effect of biocontrol agents on the induction of polyphenyl oxidase activities in chilli roots against *R. solanacearum* in glasshouse conditions; observations were recorded after 30 days of sowing. The results are expressed as average of three replications and error bars indicate standard deviation of the means. (**c**) Mean value comparison of the induction of Polyphenol oxidase activities in between chilli leaves and root tissue against *Ralstonia solanacearum* in glasshouse condition after 30 days of sowing. The results are expressed as average of three replications and error bars indicate standard deviation of the means.

Maximum PPO activity was observed in treatment T6 (1.518 ΔOD min^−1^ mg^−1^ protein) followed by T5 (1.452 ΔOD min^−1^ mg^−1^ protein). The PPO activity in leaves of chilli cv. Pusa Jwala was increased in all of the treatments at 48 h of pathogen inoculation, after which PPO activity significantly declined in all of the treatments (Figure 11a). It was noted that only rhizobacteria-treated plants showed more PPO activity than the untreated plants. Moreover, only *R. solanacearum*-treated plants also showed an enhancement of PPO activity after 48 h, i.e., 1.153 ΔOD min^−1^ mg^−1^ protein, which was significantly higher than in the untreated plants. However, *P. fluorescens* PDS1 induced higher PO activity than *B. subtilis* KA9.

(ii) Roots

Maximum PPO activity in root tissues was observed in treatment T6 (0.192 ΔOD min^−1^ mg^−1^ protein) followed by T5 treatment (0.150 ΔOD min^−1^ mg^−1^ protein). The PPO activity in chilli roots was increased in all of the treatments at 48 h of pathogen inoculation, after which PPO activity significantly declined (Figure 11b). It was noted that only rhizobacteria-treated plants showed more PPO activity than untreated plants. Moreover, only *R. solanacearum*-treated plants also showed an enhancement of PPO activity after 48 h, i.e., 0.107 ΔOD min^−1^ mg^−1^ protein, which was significantly higher than in the untreated plants. However, there was no significant difference between *B. subtilis* KA9 and *P. fluorescens* PDS1 on the induction of PPO enzyme activity.

Mean value comparison between the different treatments of the induction of polyphenol oxidase (PPO) activities in between chilli leaves and root against *Ralstonia solanacearum* in glasshouse condition revealed that treatment T6 showed a maximum induction of PPO enzyme in leaves with 1.051 and in root with the mean value of 0.113, respectively; however, the induction of this defense enzyme was found to be much higher in leave (9.30-fold) in comparison with roots (Figure 11c).

#### 2.8.3. Superoxide Dismutase (SOD)

(i) Leaves

**Figure 12:**
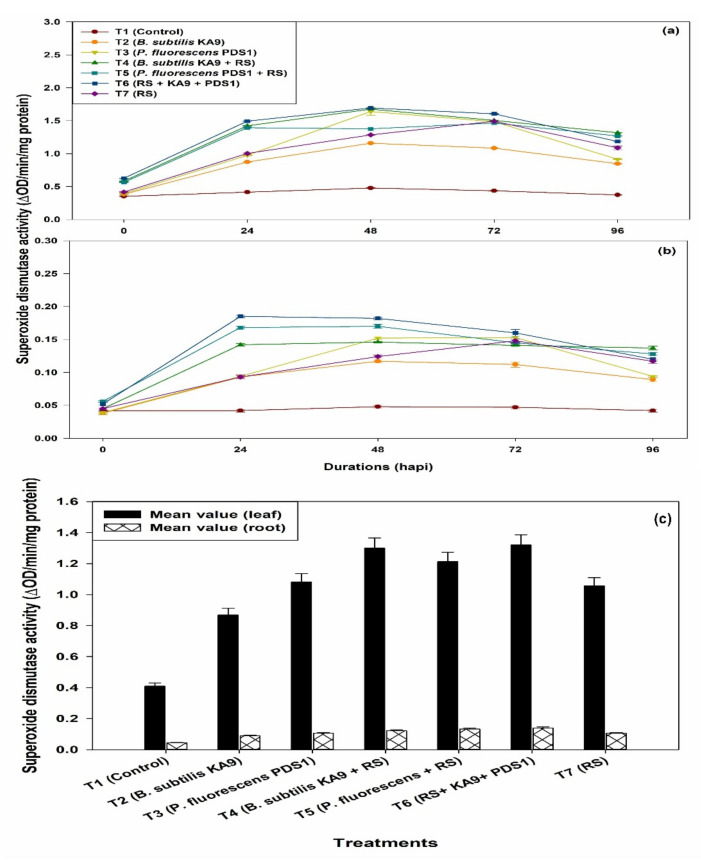
(**a**) The effect of biocontrol agents on the induction of superoxide dismutase activities in leaves of chilli crop against *R. solanacearum* in glasshouse conditions; observations were recorded after 30 days of sowing. (**b**) The effect of biocontrol agents on the induction of superoxide dismutase (SOD) activity in roots of chilli crop against *R. solanacearum* in glasshouse conditions; observations were recorded after 30 days of sowing. The results are expressed as average of three replications and error bars indicate standard deviation of the means. (**c**) Mean value comparison of the induction of superoxide dismutase activities in between chilli leaves and root tissue against *R. solanacearum* in glasshouse condition after 30 days of sowing. The results are expressed as average of three replications and error bars indicate standard deviation of the means.

In the present study, it was observed that SOD activity was increased in all of the treatments when compared with unchallenged healthy control plants. Maximum SOD activity was observed in the treatment T6 (1.693 ΔOD min^−1^ mg^−1^ protein) followed by T4 (1.675 ΔOD min^−1^ mg^−1^ protein) at 48 h. The SOD activity increased in all of the treatments at 48 h of pathogen inoculation, after which SOD activity declined significantly (Figure 12a). It was noted that only rhizobacteria-treated plants showed more SOD activity than the untreated plants. Moreover, only *R. solanacearum*-treated plants also showed an enhancement of SOD activity after 72 h, i.e., 1.492 ΔOD min^−1^ mg^−1^ protein, which was significantly higher than in the untreated plants. However, *P. fluorescens* PDS1 induced higher SOD activity than the *B. subtilis* KA9 under glasshouse conditions.

(ii) Roots

The SOD activity was increased in all of the treatments at 24 h of pathogen inoculation, after which SOD activity declined significantly (Figure 12b). It was noted that only rhizobacteria-treated plants showed more SOD activity than untreated plants. Moreover, only *R. solanacearum*-treated plants also showed an enhancement of SOD activity after 72 h, i.e., 0.148 ΔOD min^−1^ mg^−1^ protein, which was significantly higher than in the untreated plants. However, *P. fluorescens* PDS1 induced higher SOD activity in roots of chilli cv. Pusa Jwala than *B. subtilis* KA9.

Mean value comparison between the different treatments of the induction of superoxide dismutase (SOD) activities in between leaves and root tissue of chilli cv. Pusa Jwala against *R. solanacearum* in glasshouse conditions revealed that the treatment T6 showed maximum induction of SOD enzyme in leaves (1.320 ΔOD min^−1^ mg^−1^ protein) compared with root tissue (0.139 ΔOD min^−1^ mg^−1^ protein), respectively; however, the induction of this defense enzyme was found to be much higher in leaves (9.49-fold) as compared with roots (Figure 12c).

#### 2.8.4. Phenylalanine Ammonium Lyase Activity (PAL)

(i) Leaves

**Figure 13:**
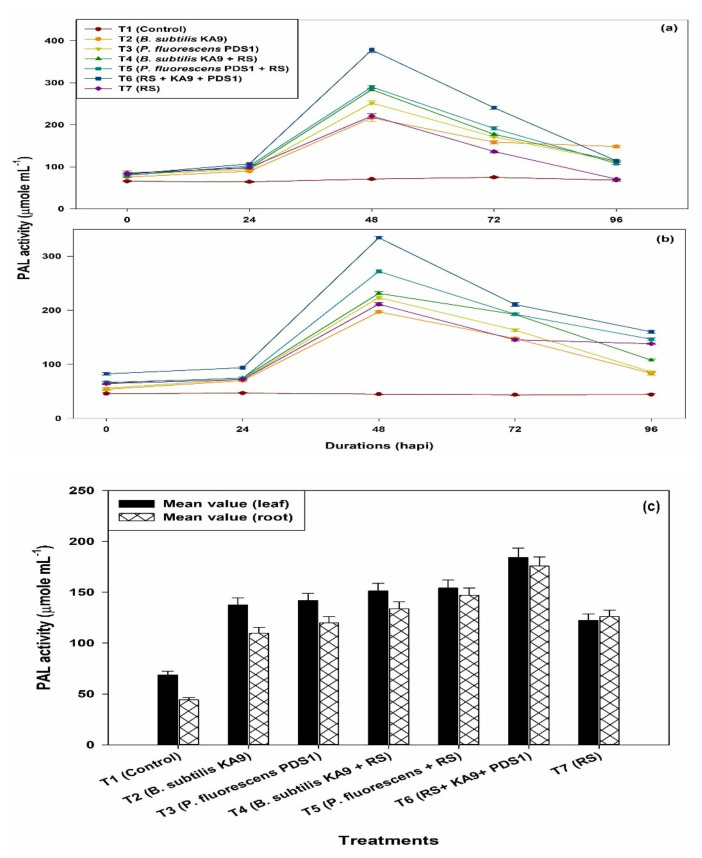
(**a**) The effect of biocontrol agents on the induction of phenyl ammonium lyase (PAL) activities in leaves of chilli crop against *R. solanacearum* in glasshouse conditions and observations were recorded after 30 days of sowing (**b**) Effect of biocontrol agents on induction of phenyl ammonium lyase activities in roots of chilli crop against *R. solanacearum* in glasshouse conditions; observations were recorded after 30 days of sowing. The results are expressed as average of three replications and error bars indicate standard deviation of the means. (**c**) Mean value comparison of the induction of phenyl ammonium lyase (PAL) activities in between chilli leaves and root tissue against *R. solanacearum* in glasshouse conditions after 30 days of sowing. The results are expressed as average of three replications and error bars indicate standard deviation of the means.

The PAL activity was increased in all of the treatments at 48 h, after which it significantly declined, to 96 h (Figure 13a). It was noted that only rhizobacteria-treated plants showed more PAL activity than untreated plants. Moreover, only *R. solanacearum*-treated plants also showed an enhancement of PAL activity after 48 h, i.e., 221.00, which was significantly higher than in the untreated plants. However, *P. fluorescens* PDS1 induced higher PAL activity than the *B. subtilis* KA9.

(ii) Roots

The PAL activity was increased in all of the treatments at 48 h, after which it significantly declined (Figure 13b). The rhizobacteria-treated plants showed more PAL activity in root tissue than untreated plants. Moreover, only *R. solanacearum*-treated plants also showed an enhancement of PAL activity after 48 h (221.33), which was significantly higher than in the untreated plants. However, *P. fluorescens* PDS1 induced higher PO activity than *B. subtilis* KA9.

Mean value comparison between the tissue of leaf and root of chilli plant in different treatments of the induction of phenyl ammonium lyase (PAL) activities in between chilli leaves and root tissue against *R. solanacearum* in glasshouse condition was studied and it was revealed that treatment T6 showed a maximum induction of PAL enzyme in leaves with 184.2 and in root with the mean value of 175.84. However, the induction of this defense enzyme was found to be slightly higher in leave tissue (1.04-fold) in comparison with root tissue (Figure 13c).

## 3. Discussion

Bacterial wilt of solanaceous crops caused by *R. solanacearum*, particularly in chilli plants, is a serious problem throughout India and it is challenging to manage the disease because of its soil-borne nature. The bacterial wilt caused by *Ralstonia solanacearum* was believed to be one of the most severe diseases, with losses of up to 90% and varying depending on the *R. solanacearum* strains, soil type, host cultivars, cropping pattern and climate [14,68,69]. Chemical pesticides are not much effective due to their pathogenic soil-borne nature. In addition, more effective and efficient biocontrol agents are needed to fulfill the rising demand for chemical-free agricultural goods. As a result, rhizobacteria with various characteristics for plant growth promotion and protection might be employed to produce novel, environmentally acceptable and effective bioformulations as a replacement for synthetic fungicides [74]. PGPR colonizes plant roots, enhancing plant growth and development through various mechanisms. The precise method through which PGPR promotes plant development is obscure; however, several mechanisms such as the production of phytohormone, the suppression of deleterious species, the activation of phosphate solubilization and increase in the absorption of mineral nutrients are often considered to play a role in plant growth promotion [75]. Several studies are available on the screening and benefits of PGPR from crop plants, especially sugarcane, maize and rice; however, very few screening studies and use of PGPR are available on chilli crops. The critical step for establishing an effective bacterial strain for disease management is to isolate and identify strong antagonistic rhizobacteria from rhizospheric soil [76]. Several rhizobacteria have been isolated from the rhizosphere of crops to treat various fungal and bacterial plant diseases [58,73,74]. *Bacillus* and *Pseudomonas* genera were dominated by various rhizobacterial antagonists, especially in the rhizospheric soil zone of tomato, chilli, brinjal and other solanaceous crops [77]. Shannon diversity was used to evaluate the diversity of the microbial communities in the chilli rhizosphere [78]. Microbial diversity was highest in the Middle Gangetic Plain region as compared with another geographical region. Similar results have been observed with microbial diversity of rhizospheric Jatropha crop, which showed to be highest in Noida and Bhopal regions rather than Jabalpur in India [79]. Similar results were observed in *Ranunculus glacialis* rhizospheric microbial diversity [80]. It has been discovered that soil microbial diversity is connected to crop health positively [81]. The findings indicate that a diversified soil microbiome (with a larger percentage of microbial species) reduces the risk of bacterial wilt epidemics. In this study, 90 soil samples were collected from rhizospheric soil of chilli crop, covering geographical coordinates ranged from latitude (13.63° N–29.57° N) and longitude (74.98° E–84.12° E). A total of 63 rhizobacteria were isolated and tested against *R. solanacearum*; 38.09% rhizobacteria showed antagonistic activity against the targeted pathogen under in vitro conditions. Similar results were found, where a total of 180 strains of *Bacillus* were isolated from the tomato rhizosphere and these strains were tested against *Pseudomonas syringae*, *Phytophthora infestans*, *Verticillium dahliae*, *Erwinia carotovora* and *Botrytis cinerea*. Moreover, 34 *Bacillus* strains were found to have the best antagonistic activity against these plant pathogens with the production of surfactin, subtilin fengycin and bacillibactin compounds [82]. Ramesh and Phadke (2012) carried out a similar study in which they isolated 109 strains of endophytic rhizobacteria from eggplants and assessed their biocontrol efficiency against *R. solanacearum* [16,83]. These 34 antagonist rhizobacteria isolates were screened for plant growth-promoting characteristics, including siderophore, IAA production, phosphate solubilization and ammonia production. Accordingly, 79.41% rhizobacterial isolates were shown to be positive in phosphate solubilization, 67.75% ammonia production, 58.82% HCN production, 55.88% siderophore production and 67.75% IAA production. This was confirmatory to earlier reports on rhizobacteria and endophytes [84,85,86]. IAA is recorded as one of the most plentiful and secreted by different genera of bacteria, such as *Rhizobium*, *Azospirillum*, *Bacillus* and *Pseudomonas* [87,88]. A similar study was conducted and found that 5 effective BN isolates showed a significant phosphorous solubilizing ability, potential to N fixation, also producing the siderophores and IAA, which could be used as indicators for a healthy growth promotion ability [89,90]. A higher level of IAA production was recorded by *Bacillus* spp. and *Pseudomonas* spp. [91,92,93,94]. Moreover, 23 bacterial isolates were identified to produce HCN and were helpful in the control of plant diseases [66]. In this study, isolates PDS1—*Pseudomonas fluorescens* (MN368159), BDS1—*Bacillus subtilis* (MN395039), UK4—*Bacillus cereus* (MT491099), UK2—*Bacillus amyloliquefaciens* (MT491100) and KA9 *Bacillus subtilis* (MT491101) were found to be the most effective PGPR that solubilized insoluble phosphorus and produced IAA, ammonia, HCN and catalase. Similar findings have been recorded in bacterial strains such as *Achromobacterium*, *Agrobacterium*, *Burkholderia*, *Aerobacter*, *Pseudomonas*, *Erwinia*, *Bacillus*, *Rhizobium*, *Microccocus* and *Flavobacterium* [95,96]. Three isolates could produce HCN: *Klebsiella pneumonia*, *Pseudomonas aeruginosa* and *Bacillus subtilis* [97,98]. According to the findings, biochemical tests for rhizobacterial identification and characterization may only be utilized to differentiate between rhizobacterial strains to a limited extent and cannot distinguish between closely related ones. Full-length 16S rRNA sequencing, on the other hand, offers information about each isolate from the chilli rhizosphere as a distinct entity. Previous research has shown I6S rRNA sequence analysis as a reliable method for studying bacterial isolates at the species level [99,100,101]. These are some of the main steps in choosing a potent biocontrol agent for plant disease management, assessing the effectiveness of antagonistic potential of biocontrol agents in suppressing plant diseases under glasshouse conditions [102]. After 40 days of PDS1 inoculation, our glasshouse screening studies revealed that chilli cv. Pusa Jwala treated by PDS1 had the lowest bacterial wilt incidence of 20.8% and the highest biocontrol efficacy 71.11%, with maximum shoot length, root length, shoot dry weight and root dry weight. The findings of glasshouse studies indicated that the tested PGPRs differed marginally in their impact on chilli disease suppression and PGP attributes; as a result, under glasshouse circumstances, all of the tested bacterial strains significantly outperformed the untreated control. Similar results showed that the lowest disease incidence of tomato bacterial wilt disease was achieved by *P. fluorescens*, followed by *P. putida* and finally *B. subtilis* and PGPR-treated plants exhibited substantial plant growth-promoting characteristics under glasshouse conditions [103,104,105].

The latest research has consistently shown that the bacterial antagonist protection enzymes against *R. solanacearum* were induced by biocontrol agents, viz., *B. subtilis* KA9 and *P. fluorescens* PDS1. Several studies have shown that several rhizobacteria can induce systemic resistance in different plants to manage soil-borne diseases. Induced systemic resistance has emerged as an essential mechanism by which certain plant growth-promoting bacteria (PGPB) prime the entire plant body to enhance protection against a wide range of pathogens. This condition of resistance is identified by the activation of latent defensive responses, which are expressed not only at the induction site but also systemically in plant parts spatially isolated from the inducer upon pathogen challenge [106]. Secondary metabolites can be produced by certain *Bacillus* members and various lytic enzymes along with the induction of systemic resistance in plants, such as increasing resistance in plants and increasing defense enzyme such as phenyl ammonia lyase, peroxidase and polyphenol oxidase activity [107]. Oxidative enzymes, such as PPO and PO, can catalyze lignin and other oxidative phenols and contribute to the formation of lignin and other oxidative phenols. Defense barriers are activated against pathogens by modifying the defense mechanism of the cell structure [108].

From the present study, the treatment with a combination of *B. subtilis* KA9 and *P. fluorescens* PDS1 applied along with pathogen *R. solanacearum* in chilli cv. Pusa Jwala plants showed higher polyphenol oxidase, peroxidase, superoxide dismutase and phenyl ammonia lyase activity, compared with 0 hour treatments and then increased at 24 h, later showed increasing trend up to 48 h, then gradually decreasing at 72 and 96 h, irrespective of all the treatments. The untreated control treatment did not show any increased level of enzyme activity. This result was supported by the findings of a study by Jayapala and his associates [107], according to which chilli seed biopriming with Bacillus sp. enhanced the defense-related enzymes such as PAL, peroxidase and polyphenol oxidase at 48 h against *Colletotrichum capsica*. Chunyu and his associates reported that *B. amyloliquefaciens* SQRT3 strain and *R. solanacearum*-treated tomato plant enhanced the polyphenol oxidase and peroxidase activities as compared with other treatments; the observations were similar to our results [109]. Ramamoorthy et al. (2002) recorded a similar result where they pretreated tomato seeds with *P. fluorescens* and showed that the bioagents induced systemic resistance against *Fusarium oxysporum* f. sp. *Lycopersici* [110]. Similarly, in another study, it was found that the biopriming of tomato seeds with rhizobacteria enhanced induced systemic resistance against *P. syringae* pv. *tomato* with increased level of peroxidase and phenyl ammonia lyase activities [111]. It was reported that jasmonic acid signaling pathway played a major role in defense mechanism against *R. solanacearum* in tomato. When tomato plant treated with *B. subtilis* and challenged pathogen *Erwinia carotovora* subsp. *carotovora* enhanced the phenyl ammonia lyase and superoxide dismutase activities in tomato plant against the challenged pathogen, these findings supported the result of the present study [112,113]. Increased SOD leads to the deposition of more H_2_O_2_, which is necessary for plant resistance against pathogen [114]. Most of these findings are consistent with current observations in which the enhancement of defensive enzymes such as PAL, PPO, POD and SOD has been seen to be involved in the inhibition of plant disease [88]. Our results were on par with the findings of Vanitha and her associates [115], who reported that tomato seedlings pretreated with *P. fluorescens* enhanced the polyphenol oxidase, peroxidase, lipoxygenase and phenyl ammonia lyase activities after the inoculation of *R. solanacearum*. 

## 4. Materials and Methods

### 4.1. Isolation of Rhizobacteria from Rhizospheric Soil of Chilli Plants

During the research period of 2013–2019, surveys were made for the collection of rhizospheric soil of chilli crop from different locations of India such as Haldwani, Pantnagar, Chorgaliya and Chaffi (Uttarakhand), Gonad, Faizabad, Mau (Uttar Pradesh), Bhojpur, Buxar (Bihar), Dharwad, Gulbarga, Raichur, (Karnataka), Warangal Guntur, (Andhra Pradesh) and under western Himalayan region, Lower Gangetic Plain, Middle Gangetic plain, Upper Gangetic Plains, Eastern Plateau and Hills and Southern Plateau and Hills regions. The samples were brought to the laboratory, Division of Plant Pathology, ICAR—Indian Agricultural Research Institute, New Delhi, for plant growth-promoting rhizobacteria characterization, isolation and identification.

The rhizospheric soil samples collected from the field (Table 1) were taken, about 10 grams and mixed well in a 250 mL flask containing 100 mL of sterilized distilled water and then heated to 60 °C for 15 min. After that, up to 10^−5^ of soil suspension was prepared for serial dilution. The 100 µL of an aliquot from 10^−3^ and 10^−5^ serial dilution were poured onto the Petri plates containing CPG (casamino peptone glucose), NA (nutrient agar), TSA (tryptone soya agar) and Kings’ B media [77] and spread by sterile L-shaped glass spreader. The Petri plates were incubated at 28 ± 1 °C for 48–72 h to allow bacteria to grow onto the medium. The single bacterium colony was selected and transferred to the slanting YGCA (yeast glucose carbonate agar). The cultures were kept on the slant of the YGCA and stored for further use at 4 °C.

### 4.2. Shannon Index of Microbial Diversity

The Shannon microbial diversity index was calculated by phenotyping the different chilli-rhizobacteria colonies cultured on growth media, *viz*., KB, NA, CPG and TSA agar plates. On randomly grown agar plates containing 20–300 bacterial colonies, 63 rhizobacteria colonies were selected for colony morphology analysis. The size, shape, color, elevation, surface and margin of each colony were given a code. The Shannon Diversity Index (H’) is defined as H’= −pilog2pi, where pi is the number of individuals divided by the total number of isolates in the sample under examination.

### 4.3. Antagonistic Activity of Rhizobacteria against R. solanacearum In Vitro Conditions

The dual culture approach was used as previously described by Singh et al. (2016) [116]. In vitro, this method was used to evaluate the antagonistic potential of 63 chilli rhizobacterial isolates against *R. solanacearum* UTT25. The Plant Pathology Division, IARI, New Delhi, provided the strain *R solanacearum* UTT25. The bacteria were cultured on a nutrient broth medium at 30 ± 1 °C for 48 h and the population of bacteria was maintained constant (0.1 OD600). A measure of 100 μL of *R. solanacearum* UTT25 culture was poured onto Petri plates containing casein peptone glucose agar (CPG) medium to create a lawn. Then, three 0.5 cm diameter wells in each Petri plate were created using a sterilized cork borer. The 40 µL culture of rhizobacteria isolates cultured in nutrient broth containing 0.1 OD at 600 nm was put into each well independently. The inhibition zone generated by rhizobacterial isolates was measured after 48 h of incubation at 30 ± 1 °C. These isolates were chosen for further research, which formed an inhibition zone with a diameter of more than 0.5 cm.

### 4.4. Morphological and Biochemical Analysis 

Biochemical tests further characterized 63 isolates as per methodology described in Bergey’s Manual of Systematic Bacteriology [117]. These tests include gelatine liquefication, starch hydrolysis, H_2_S, arginine hydrolase, KOH, catalase, citrate and fermentation of various sugars. 

### 4.5. PGPR Characteristics of Rhizospheric Bacteria

#### 4.5.1. Phosphorus Solubilization Assay

A quick screening of phosphate solubilization by rhizobacteria was carried out on Pikovaskaya media [118]. The 48 h old culture of most likely antagonistic isolates was injected and incubated at 28 °C for 3–5 days in the Pikovaskaya (PVK) broth medium. Then, in a separate growth tube, 1 mL of each rhizobacterial culture was recovered and 10 mL of ammonium molybdate was added to each bacterial culture, which was thoroughly mixed. The blue color intensity of the solution at a wavelength of 600 nm was measured using a UV-VIS spectrophotometer (Hitachi, U-2900) and the corresponding quantity of soluble phosphorous was calculated using the standard curve [119].

#### 4.5.2. Indole Acetic Acid (IAA) Production Assay

Vikram et al. (2007) devised a quantitative method to quantify indole acetic acid production by selected rhizobacterial strains [120]. In a 100 mL conical flask, 25 mL of the supernatant of the most potent 34 chilli-rhizobacterial isolates were collected and we adjusted the pH to 2.8 with 1 N HCl. We added appropriate amounts of diethyl ether to the reaction mixture, which had incubated in the dark for 4 h. In a different funnel, diethyl ether would be used to extract indole acetic acid at 4 °C. The organic phase was separated and the solvent was collected to determine the amount of IAA in the methanol extract. Subsequently, 0.5 mL of methanol extract, 1.5 mL of double distilled water and 4 mL of Sapler’s reagent (1 mL of 0.5 M FeCl_3_ in 50 mL of 35% perchloric acid) were added and incubated in the dark for 1 hour. In a spectrophotometer, the intensity of the pink color produced was measured at 535 nm. The amount of IAA in the supernatant was calculated using a standard curve with a known concentration of IAA (Sigma–Aldrich, Germany) and represented as µg/25 mL of medium.

#### 4.5.3. Siderophore Production Assay

Schwyn and Neiland’s method [121] of siderophore production of rhizobacteria isolates using CAS agar solution plates assay was applied. The production of siderophore was studied in Petri dishes using CAS-agar. Then, 48-hour-old rhizobacteria isolate cultures were impaled with sterilized toothpicks on CAS-agar plates and cultivated in the dark for two weeks at 28 °C. Siderophore strains were defined as colonies with orange zones. In solid media, the experiments were performed in triplicate. Control plates of CAS-agar (uninoculated control) were grown and incubated for 1–14 days under the same circumstances, but no color change in CAS-blue agar was observed.

#### 4.5.4. Ammonia Production Assay

The rhizobacteria isolates were grown in autoclaved 4% peptone water and incubated for 4 days at 30° C. After incubation, 1 mL of Nessler’s reagent was added to the tube containing bacterial suspension. A pale-yellow tint suggests a minimal level of ammonia production, whereas a deep yellow to brownish tint suggests maximal ammonia production. A spectrophotometer was used to test the absorbance at 450 nm. Furthermore, the amount of ammonia produced by the rhizobacterial isolates was quantitatively estimated and compared to a standard curve created using a standard ammonium sulphate solution.

#### 4.5.5. HCN Production Assay

Following the procedure outlined by Bakker and Schipper [122], all isolated rhizobacteria were evaluated for hydrogen cyanide generation. On glycine-added nutrient agar media (4.4 g/L), each rhizobacterium was streaked. A Whatman number 1 filter paper was used to cover the agar, which had been previously soaked in a particular solution (0.5% picric acid and 2% sodium carbonate w/v). The plates were sealed with Parafilm and left to incubate for 48 h at 28 °C. A shift in filter paper color from yellow to light brown, brown, or reddish-brown was recorded as a positive (+) and negative (−) response.

### 4.6. 16S rRNA Gene-Based Identification

#### Bacterial Genomic DNA Extraction and 16S rRNA PCR

CTAB technique was used to extract genomic DNA from bacterial isolates obtained from various locations of India [123]. For 16S rRNA-based PCR identification, genomic DNA was taken from five rhizobacteria: PDS1, UK4, BDS1, UK2 and KA9. A set of strain-specific forward and reverse primers were utilized to amplify 1500 bp of the rRNA gene fragment. The PCR amplification was carried out in a 50 µl reaction containing 25 µl of 2X PCR Master Mix (Thermo Fisher Scientific), 1µL of template DNA (0.5 g), 0.2 µL of both forward and reverse primers (2.5 M of each) and 24.6 µL of nuclease-free water in a PCR tube added in that order in a C-1000 Thermal Cycler (Model C-1000, Bio-rad). An initial denaturation phase of 5 min at 95 °C was followed by 30 cycles of 1 min at 94 °C, 56 °C for 1 min and 72 °C for 2 min and a final extension step of 5 min at 72 °C. Electrophoresis of PCR products (amplicons) on a 1% agarose TAE gel containing ethidium bromide was performed and observed using a gel documentation system (BIO-RAD, GEL DOCTM XR+ with LabTM imaging software) (http://www.scigenom.com, accessed on 10 June 2021), using both forward and reverse primers to sequence purified PCR aliquots of these strains. The purified PCR products were bidirectionally sequenced through the Sanger sequencing method (Scigenom Pvt Ltd., Banglore, India) to obtain the maximum length. Bio Edit Version 7.0.5 was used to analyze and assemble the sequences. Multiple sequence alignment was performed for all 16S rRNA gene sequences, using Clustal-W through MEGA6 software [124]. For all of the isolates, aligned sequences were used in the BLAST interface of the NCBI database (http://blast.ncbi.nlm.gov, accessed on 17 July 2021) and the similarity was confirmed by identity percentage. Following identification, all aligned sequences of the isolates were submitted to NCBI GenBank for an accession number. The molecular phylogenetic analysis was conducted by using the maximum-likelihood method. Mega 6.06 software was used to infer the evolutionary history or consensus tree using the maximum-likelihood method with Taimura Nei Model [124]. The bootstrap method was used to verify nodal resilience and replication of 1000 was used to determine phylogenetic robustness. The cladogram was constructed to explain the relationship between the isolates and the different species studied.

### 4.7. Evaluation of Growth-Promoting Ability under Glasshouse Experiment 

*Pseudomonas fluorescens* (PDS1) and *Bacillus subtilis* (KA9) rhizobacterial isolates were grown in sterile NB agar plates at 28 ± 1 °C for 48 h. The cells were diluted to 10^8^ CFU/mL in sterile distilled water. The pathogenic bacteria *R. solanacearum* UTT25 was grown for 48 h at 28 °C on CPG agar medium and maintained an inoculum load of 10^8^ CFU/mL by using sterile distilled water. Chilli cv. Pusa Jwala seeds were sown in plastic trays in a Phytotron at 28 ± 2° C with a 2:1:1 mixture of peat mass, vermiculite, sand and watered daily. Twenty-five-day-old chilli seedlings were transferred into six-inch autoclave earthen pots containing 1 kg of soil mixture. Five days after transplantation, the plants were concurrently treated with 5 mL suspension (10^8^ CFU/mL) of rhizobacteria and *R. solanacearum* UTT25 at root zone of plant. One set of negative control inoculated only with *R. solanacearum* UTT25 was also maintained with three replications. The 11 treatments were included as T1 (BDS1), T2 (KA3), T3 (KA9), T4 (UK2), T5 (BR3), T6 (UP13), T7 (AP2), T8 (PDS1), T9 (UK4), T10 (DL3) and T11 (Control). The plants were tested to determine wilt intensity as well as fresh and dry weight. The severity of the wilt was studied. The average percentage of wilt for each treatment was calculated. The following scale was used to rate the severity of the disease; 1: no symptoms; 2: one wilted leaf; 3: two to three wilted leaves; 4: four or more wilted leaves; 5: entire plant wilted (dead plant). The wilt incidence was calculated after 30 days of inoculation using the following formula [125].

Wilt intensity (%) (I) = [∑ (n_i_ × v_i_) ÷ (V × N)] × 100

where, n_i_ = number of plants with respective disease rating; v_i_ = disease rating (1, 2, 3, 4, or 5); V = the highest disease rating; and N = the number of plants observed. The dry weight of the root and shoot of the plant was used to determine growth-stimulating effectiveness, as described by Singh et al. (2016) [116].

### 4.8. Evaluation of Biochemical Defensive Enzymes under Glasshouse Experiment

For determination of defense enzyme studies in response to Rhizobacteria-treated chilli plants under glasshouse conditions, about 0.5 g leaf tissue of chilli cv. Pusa Jwala was collected from each treatment for enzyme extraction at intervals of 0, 24, 48, 72 and 96 h for each biochemical assay. Estimation of SOD activity was recorded with the method described by Beauchamp and Fridovich [126]; the polyphenol oxidase (PPO) activity was recorded by the method described by Mayer [127]; the peroxidase activity was measured using the method described by Hammerschmidt [128]; and the PAL activity was measured using the method described by Amrhein [129].

### 4.9. Statistical Analysis

Data from laboratory and glasshouse experiments were analyzed by using one-way analysis of variance followed by LSD test and biochemical analysis of rhizospheric bacteria was analyzed by one-way analysis of variance followed by Duncan’s multiple range test (DMRT) using Statistical Product and Service Solution (SPSS) version 16.0 software Developed by IBM SPSS, Chichago, Illinois. The treatment mean values were compared with LSD test and Duncan’s multiple range tests at *p* ≤ 0.05 significance level. The values in laboratory conditions and glasshouse experiments were shown in figures as the mean of three replications ± standard deviation.

## 5. Conclusions

Through an in vitro investigation, the bacterial strains that demonstrated antagonistic potential against *R. solanacearum* were characterized for PGP traits, phytoharmones, HCN, catalase, indole-3-acetic acid (IAA) and siderophore production and we found five strains having the best potential to produce all PGP traits with the potent antagonistic characteristics. The 16S rRNA sequencing analysis reveals that these best five strains were assigned to PDS1—*Pseudomonas fluorescens*, BDS1—*Bacillus subtilis*, UK4—*Bacillus cereus*, UK2—*Bacillus amyloliquefaciens* and KA9—*Bacillus subtilis*. In glasshouse trials, rhizobacterial strains were found to be effective in managing wilt disease along with enhancement in chilli growth parameters. These findings demonstrated the relevance of natural rhizobacteria in the control of soil-borne plant pathogenic bacteria and the possibility of using *Bacillus* and *Pseudomonas* spp. to make bio-fertilizers and biopesticides. The findings support the use of antagonistic rhizobacteria (PGPR) formulations on a field scale as a safe and environment-friendly application to protect the chilli crop from bacterial wilt disease and increase its productivity. The induction of defensive enzymes PAL, PPO, SOD and PO by *Pseudomonas fluorescens* PDS1 and *Bacillus subtilis* KA9 in chilli leaf and root tissues might have collectively contributed to induced resistance in chilli plants against *Ralstonia solanacearum*. This finding supports the view of the plant growth-promoting rhizobacteria boosting defense-related enzymes and limiting pathogen growth in chilli plants, respectively, hence managing the chilli bacterial wilt.

## Figures and Tables

**Table 1:** The location of chilli rhizospheric soils collected from the sampling sites of different agro-climatic regions of India.

Agro-Climatic Region	Place of Collection	Geographical Coordinates		Location	Source of Isolation	Host		Annual Pluviometry (mm)	Cultivars of Chilli	Year of Collection
		Latitude	Longitude							
Upper Gangetic Plains Region	Saharanpur (Uttar Pradesh)	29.57° N	77.34° E	India	Rhizospheric soil	Chilli	21	750–1500	Jawala Pant C-1, Pahadi, Kalyanpur	2014–15
	Kannauj (Uttar Pradesh)	27.04° N	79.91° E	India	Rhizospheric soil	Chilli				
	Loni (Uttar Pradesh)	28.72° N	77.30° E	India	Rhizospheric soil	Chilli				
	Pisawa (Uttar Pradesh)	28.11° N	77.76° E	India	Rhizospheric soil	Chilli				
	Udham Nagar (Uttarakhand)	28.95° N	79.48° E	India	Rhizospheric soil	Chilli				
Western Himalayan Region	Golapar (Uttarakhand)	29.17° N	79.59° E	India	Rhizospheric soil	Chilli	16	750–1500	PusaSadabahar, Pusa Jwala and Pant C-1	2018–19
	Chargaliya (Uttarakhand)	29.12° N	79.70° E	India	Rhizospheric soil	Chilli				
	Chaffi (Hill region of Uttarakhand,)	29.39° N	79.55° E	India	Rhizospheric soil	Chilli				
Middle Gangetic Plain Region1	Mau (Eastern Uttar Pradesh)	25.89° N	83.48° E	India	Rhizospheric soil	Chilli	25	1000–2000	MotiMirchi, Chittee, NP-46, Unknown	2014–15
	Varanasi (Eastern Uttar Pradesh)	25.44° N	82.85° E	India	Rhizospheric soil	Chilli				
	Bhojpur (Bihar)	25.58° N	84.12° E	India	Rhizospheric soil	Chilli				
	Buxar (Bihar)	25.55° N	83.95° E	India	Rhizospheric soil	Chilli				
Trans-Ganga Plains Region	Delhi	28.64° N	77.16° E	India	Rhizospheric soil	Chilli	5	650–1250	Pusa Jwala	2018–19
	Haryana	29.96° N	76.89° E	India	Rhizospheric soil	Chilli				
Southern Plateau and Hills region	Warangal (Telangana)	17.93° N	79.57° E	India	Rhizospheric soil	Chilli	23	500–1000	Maduru, Karakulu, Sannalu, Jawala, Bayadgi	2014–15
	Guntur (Andhra Pradesh)	16.32° N	80.45° E	India	Rhizospheric soil	Chilli				
	Raichur (Karnataka)	16.22° N	77.36° E	India	Rhizospheric soil	Chilli				
	Chikkamagaluru, (Karnataka)	13.63° N	75.73° E	India	Rhizospheric soil	Chilli				
	Dharwad (Karnataka)	15.43° N	74.98° E	India	Rhizospheric soil	Chilli				

**Table 2:** Biochemical analysis of rhizospheric bacteria isolated from chilli rhizosphere collected from different agroclimatic zones.

Agroclimatic Zone Region	Strains	Phosphate Solubilization		Indole-3-Acetic Acid	Siderophore	Ammonia	HCN	Root Colonization
		HD (mm)	mg/mL	µg/mL	Q	(mol/mL)	Q	
Southern Plateau and Hills region	KA2	5.73 ± 0.32 ^gh^	3.89 ± 0.12 ^cd^	2.53 ± 0.15 ^fg^	+	4.59 ± 0.16 ^ab^	+	+
	KA3	8.13 ± 0.24 ^cde^	4.89 ± 0.11 ^b^	2.32 ± 0.12 ^g^	−	2.82 ± 0.25 ^e^	−	+
	KA5	3.89 ± 0.37 ^ijk^	1.35 ± 0.03 ^ghijk^	0.000	+	1.70 ± 0.15 ^f^	+	+
	KA7	0.000	0.000	3.52 ± 0.22 ^de^	−	2.44 ± 0.22 ^e^	−	+
	KA9	13.40 ± 0.61 ^a^	5.92 ± 0.07 ^a^	5.65 ± 0.19 ^b^	+	4.80 ± 0.09 ^bc^	+	+
	KA13	0.08 ± 0.01	0.93 ± 0.02 ^ijkl^	3.09 ± 0.10 ^ef^	−	0.000	−	+
	KA14	4.25 ± 0.30 ^ij^	1.54 ± 0.047 ^ghij^	2.49 ± 0.18 ^fg^	+	2.58 ± 0.20 ^e^	−	+
	KA15	8.25 ± 0.21 ^cde^	3.63 ± 0.26 ^cd^	0.000	+	0.000	+	+
	KA17	0.05 ± 0.02	0.87 ± 0.03 ^ijkl^	3.72 ± 0.16 ^de^	−	2.67 ± 0.17 ^e^	−	+
	AP8	7.83 ± 0.32 ^def^	4.19 ± 0.23 ^bc^	2.81 ± 0.20 ^fg^	+	1.84 ± 0.08 ^f^	−	+
	AP13	9.31 ± 0.32 ^c^	4.74 ± 0.27 ^b^	4.89 ± 0.17 ^c^	−	1.85 ± 0.04 ^f^		+
	AP2	0.040 ± 0.017	0.70 ± 0.10 ^ijkl^	0.000	+	2.45 ± 0.13 ^e^	−	+
	AP6	7.760 ± 0.378 ^def^	3.75 ± 0.32 ^cd^	3.72 ± 0.14 ^de^	−	1.57 ± 0.16 ^f^	−	+
	AP11	3.403 ± 0.265 ^jk^	1.51 ± 0.03 ^ghij^	3.77 ± 0.28 ^d^	−	0.000	+	+
Western Himalayan Region	UK2	10.64 ± 0.58 ^b^	4.64 ± 0.24 ^b^	2.83 ± 0.20 ^fg^	+	4.98 ± 0.08 ^ab^	+	+
	UK6	8.73 ± 0.31 ^cd^	3.93 ± 0.30 ^cd^	0.000	−	0.000	−	+
	UK4	12.53 ± 0.66 ^a^	5.78 ± 0.24 ^a^	2.65 ± 0.35 ^fg^	+	4.37 ± 0.16 ^c^	+	+
	UK7	3.34 ± 0.52 ^ijk^	1.92 ± 0.13 ^gh^	2.76 ± 0.22 ^fg^	+	2.78 ± 0.19 ^e^	−	+
Upper Gangetic Plains Region	UK8	3.77 ± 0.24 ^ijk^	1.78 ± 0.06 ^gh^	0.000	−	0.000	+	+
	UP2	9.10 ± 0.06 ^c^	4.73 ± 0.26 ^b^	2.52 ± 0.22 ^fg^	−	2.46 ± 0.18 ^e^	+	+
	BDS1	12.74 ± 0.39 ^a^	5.84 ± 0.26 ^a^	4.92 ± 0.13 ^c^	+	2.42 ± 0.16 ^e^	+	+
	UP3	2.87 ± 0.36	1.24 ± 0.11 ^hijk^	2.63 ± 0.19 ^fg^	+	2.4 ± 0.14 ^e^	+	+
	BDS2	9.30 ± 0.32 ^c^	4.76 ± 0.23 ^b^	0.000	−	0.000	+	+
	BR3	8.81 ± 0.46 ^cd^	2.69 ± 0.22 ^f^	2.75 ± 0.28 ^fg^	+	0.000	+	+
	BR4	7.22 ± 0.34 ^ef^	2.72 ± 0.32 ^f^	2.673 ± 0.23 ^fg^	+	4.33 ± 0.12 ^c^	+	+
Middle Gangetic Plain Region	BR5	7.61 ± 0.69 ^def^	3.40 ± 0.42 ^de^	0.000	+	0.000	+	+
	PDS1	13.39 ± 0.50 ^a^	5.77 ± 0.28 ^a^	7.26 ± 0.32 ^a^	++	4.70 ± 0.27 ^ab^	+	+
	UP13	0.000	0.000	5.55 ± 0.28 ^b^	+	3.57 ± 0.19 ^d^	+	+
	PDS3	11.20 ± 0.67 ^b^	4.74 ± 0.23 ^b^	0.000	−	0.000	+	+
	UP7	3.49 ± 0.35 ^jk^	1.59 ± 0.25 ^ghi^	0.000	+	0.000	−	+
Trans-Ganga Plains Region	DL3	4.67 ± 0.50 ^hi^	1.97 ± 0.13 ^g^	0.000	−	3.81 ± 0.21 ^d^	−	+
	DL5	6.67 ± 0.32 ^fg^	3.157 ± 0.386 ^ef^	2.747 ± 0.226 ^fg^	+	5.33 ± 0.21 ^a^	+	+
	DL1	0.01 ± 0.01	0.523 ± 0.187 ^l^	2.840 ± 0.149 ^fg^	−	0.000	+	+
	DL8	0.000	0.000	0.000	−	2.84 ± 0.07 ^e^	−	+

Values given in the column are the average of three replications followed by standard deviation. Values with different alphabetical (a–l) superscripts within a column are significantly different (*p*  ≤ 0.05) using Duncan’s multiple range tests (DMRT). Key: + = positive, − = negative.

**Table 3:** Evaluation of potential antagonistic rhizobacteria on growth promotion of chilli cv. Pusa Jwala at 30 days after inoculation under glasshouse condition.

Strains Treatment/Growth Trait	Wilt Disease Incidence (%)	Biocontrol Efficacy (%)	Shoot Length (cm)	GPE (%)	Shoot Fresh Weight (g)	GPE (%)	Shoot Dry Weight (g)	GPE (%)	Root Length (cm)	GPE (%)	Root Fresh Weight (g)	GPE (%)	Root Dry Weight (g)	GPE (%)
BDS1	29.70 ^i^	58.75 ^d^	15.60 ^d^	26.82	8.50 ^c^	37.09	0.90 ^c^	55.17	9.61 ^c^	36.89	1.26 ^d^	28.57	0.24 ^d^	26.31
KA3	38.20 ^f^	46.94 ^f^	14.20 ^g^	15.44	7.10 ^f^	14.51	0.82 ^d^	41.37	8.16 ^f^	16.23	1.06 ^g^	8.16	0.21 ^f^	10.52
KA9	24.50 ^j^	65.97 ^b^	18.80 ^b^	52.84	9.50 ^b^	53.22	1.08 ^b^	86.20	11.46 ^b^	63.24	1.67 ^b^	70.40	0.27 ^b^	42.10
UK2	28.80 ^h^	60.00 ^c^	16.10 ^c^	30.89	7.70 ^e^	24.19	0.90 ^c^	55.17	9.82 ^c^	39.88	1.39 ^c^	41.83	0.22 ^e^	15.78
BR3	41.80 ^e^	41.94 ^g^	14.80 ^e^	20.32	6.90 ^g^	11.29	0.78 ^e^	34.48	8.19 ^g^	16.66	1.09 ^f^	11.22	0.20 ^g^	5.26
UP13	47.20 ^c^	34.44 ^i^	14.50 ^f^	17.88	6.60 ^h^	6.45	0.71 ^f^	22.41	8.21 ^e^	16.95	1.08 ^f^	10.20	0.21 ^f^	10.52
AP2	42.40 ^d^	41.10 ^h^	15.60 ^d^	26.82	7.90 ^d^	27.41	0.82 ^d^	41.37	9.42 ^d^	34.18	1.17 ^e^	19.38	0.25 ^c^	31.57
PDS1	20.80 ^k^	71.11 ^a^	19.70 ^a^	60.16	10.50 ^a^	69.35	1.13 ^a^	94.82	12.2 ^a^	73.78	1.89 ^a^	92.85	0.31 ^a^	63.15
UK4	34.60 ^g^	51.93 ^e^	13.20 ^h^	7.31	6.60 ^h^	6.45	0.69 ^g^	18.96	7.18 ^i^	2.27	0.99 ^i^	1.02	0.21 ^f^	10.52
DL3	50.00 ^b^	30.56 ^j^	13.40 ^i^	8.94	6.50 ^i^	4.83	0.69 ^g^	18.96	7.30 ^h^	3.98	1.03 ^h^	5.10	0.22 ^e^	15.78
Control	72.00 ^a^	-	12.30 ^j^	-	6.20 ^j^	-	0.58 ^h^	-	7.02 ^j^	-	0.98 ^j^		0.19 ^h^	-

Values given in column are the average of three replications. Values with different alphabetical (a–k) superscripts within a column are significantly different as determined by LSD test (α = 0.05).

**Table 4:** Characterization of best five rhizobacterial isolates from the chilli rhizosphere in the hilly regions of Uttarakhand and Central plain of Karnataka, India.

Characters	Rhizobacteria				
	*Pseudomonas fluorescens* Strain PDS1	*Bacillus subtilis* Strain BDS1	*Bacillus cereus* Strain UK4	*Bacillus* *amyloliquefaciens* Strain UK2	*Bacillus subtilis* Strain KA9
Accession no.	MN368159	MN395039	MT491099	MT491100	MT491101
Morphological characters					
Gram’s reactions	Negative	Positive	Positive	Positive	Positive
Pigmentation	Cream white	Gray-white	White	Light brown	Gray-white
Appearance	Rod-shaped	Bacilli	Bacilli	Bacilli	Bacilli
Colony characters					
Size	Small	Medium	Medium	Medium	Medium
Shape	Uniform	Round	Round	Round	Round
Margin	Entire round	Thick ridges	Undulate	Entire round	Thick ridges
Opacity	Opaque	Opaque	Opaque	Semi-transparent	Opaque
Elevation	Convex	Convex	Convex	Convex	Convex
Texture	Smooth, shiny	Smooth, moist	Irregular	Smooth	Smooth, moist
Biochemical characters					
H_2_S	+	−	+	−	+
Arginine	+	+	+	+	+
Catalase	+	+	+	+	+
Amylase	+	+	+	+	+
Glucose	+	+	+	+	+
Galactose	−	−	−	−	−
Lactose	−	−	−	−	−
Sorbitol	−	−	−	−	−
Mannose	+	+	+	+	+
Xylose	+	+	+	+	+
Sucrose	+	+	+	+	+
Citrate	+	+	+	+	+
PGP traits					
IAA Production	+	++	+	+	+++
Phosphorus Solubilization	+	+	+	+	+
Ammonia Production	++	+	++	++	++
HCN Production	−	−	−	−	−
Siderophore Production	++	+	+	+	+

Key: + = positive, ++ = moderately positive, +++ = strongly positive, − = negative; the representative results of three separate assays are shown.

## Data Availability

Not applicable.

## Supplementary Materials

The following are available online at www.mdpi.com/article/10.3390/plants10102125/s1, Table S1: Characterization of rhizobacteria isolates on the basis of colonies morphology isolated from rhizospheric soil of chilli from different agro-climatic regions of India, Table S2: Abundance and diversity index of rhizobacterial population isolated from rhizospheric soil on different microbiological media.
