# Supplementary material for: Screening and Biocontrol Potential of Rhizobacteria Native to Gangetic Plains and Hilly Regions to Induce Systemic Resistance and Promote Plant Growth in Chilli against Bacterial Wilt Disease
Source: Plants (Basel). 2021 Oct 7;10(10):2125. doi: 10.3390/plants10102125 (PMC8541367; doi:10.3390/plants10102125)
Supplement: Supplementary file 1 [file plants-10-02125-s001.zip › Supplementary Table S1.pdf]

Supplementary Table S1. Characterization of rhizobacteria isolates on the basis of colonies morphology isolated from rhizospheric soil of chilli from different agro-climatic regions of India.

Isolates	Size	shape	Margin	Opacity	Elevation	Texture	Pigmentation
KA2	Large	Round	Entire round	Opaque	Convex	Smooth	Off white
KA3	Large	Round	Entire round	Opaque	Concave	Smooth	Creamy-white
KA5	Medium	Round	Entire round	Transparent	Flat	Smooth	-
KA7	Large	Irregular	Filamentous	Opaque	Flat	Rough	Off-white
KA9	Medium	Round	thick ridges	opaque	Convex	smooth, moist	Gray-white
KA13	Large	Uniform	Undulate	Opaque	Flat	Rough	White
KA14	Large	Regular	Entire round	Opaque	Convex	Smooth	Greenish yellow
KA15	Small	Regular	Entire round	Opaque	Convex	Smooth	Light red
KA17	Small	Irregular	Entire round	Opaque	Convex	Smooth	Light yellow
AP8	Large	Round	Undulate	Semi-transparent	Flat	Slummy -rough	Off-white
AP13	Large	Irregular	Undulate	Opaque	Flat	Smooth	Greenish yellow
AP2	Small	Round	Entire round	Opaque	Convex	Smooth	White
AP6	Large	Irregular	Entire round	Opaque	Flat	Smooth	Pale yellow
AP11	Small	Uniform	Entire round	Transparent	Convex	Smooth-slums	-
UK2	Medium	Uniform	Entire round	Semi-transparent	Flat	Smooth	Light brown
UK6	Very small	Uniform	Entire round	Opaque	Flat	Smooth	Greenish yellow
UK4	Medium	Irregular	Undulate	Opaque	Flat	Irregular	White
UK7	Medium	Uniform	Entire round	Opaque	Flat	Smooth-shiny	Off-white
UK8	Large	Uniform	Entire round	Semi-transparent	Flat	Smooth-shiny	-
UP2	Small	Uniform	Entire round	Opaque	Convex	Smooth	Off- white
BDS1	Medium	Round	thick ridges	opaque	Convex	smooth, moist	Gray-white
UP3	Very small	Uniform	Entire round	Opaque	Flat	Smooth	White
BDS2	Medium	Round	Entire round	Transparent	Flat	Smooth	-
BR3	Small	Uniform	Entire round	Transparent	Convex	Smooth-slums	-
BR4	Large	Regular	Entire round	Opaque	Convex	Smooth	Off white
BR5	Large	Round	Entire round	Opaque	Convex	Smooth	Off white
PDS1	Small	Uniform	Entire round	Opaque	Convex	Smooth, Shiny	Greenish yellow
UP13	Large	Regular	Entire round	Opaque	Convex	Smooth	Off white
PDS3	Large	Round	Entire round	Opaque	Convex	Smooth	Off white
UP7	Very small	Uniform	Entire round	Opaque	Flat	Smooth	White
DL3	Small	Uniform	Entire round	Transparent	Convex	Smooth-slums	-

DL5	Large	Regular	Entire round	Opaque	Convex	Smooth	Off white
DL1	Medium	Round	Entire round	Transparent	Flat	Smooth	-
DL8	Small	Uniform	Entire round	Semi-Transparent	Convex	Smooth-slums	Greenish yellow
