# Supplementary material for: Screening and Biocontrol Potential of Rhizobacteria Native to Gangetic Plains and Hilly Regions to Induce Systemic Resistance and Promote Plant Growth in Chilli against Bacterial Wilt Disease
Source: Plants (Basel). 2021 Oct 7;10(10):2125. doi: 10.3390/plants10102125 (PMC8541367; doi:10.3390/plants10102125)
Supplement: Supplementary file 1 [file plants-10-02125-s001.zip › Supplementary Table S2.pdf]

SupplementaryTable S2. Abundance and diversity index of rhizobacterial population isolated from rhizospheric soil on different microbiological media

	**Rhizobacterial Population Avg.log (Cfu/g of soil)**											
**Agro-climatic regions**	**Nutrient Agar**	**Number of bacteria types (n)**	**Shannon diversity index**	**Kings' B**	**Number of bacteria types (n)**	**Shannon diversity index**	**Tryptone soya agar**	**Number of bacteria types (n)**	**Shannon diversity index**	**Casamino peptone glucose (CPG) media**	**Number of bacteria types (n)**	**Shannon diversity index**
Upper Gangetic Plains Region	9.64 ± 0.14^{a}	9.00 ±1.00	0.23 ± 0.08	9.33 ± 0.09^{c}	2.00 ± 0.00	0.08 ± 0.03	9.47 ± 0.44^{b}	5.00 ± 1.00	0.16 ± 0.66	9.15± ± 0.72^{d}	2.00 ±0.00	0.08 ± 0.03
Western Himalayan Region	9.58 ± 0.09^{c}	8 .00± 0.00	0.22 ± 0.12	9.23 ± 0.16^{d}	1.00 ± 1.00	0.05 ± 0.10	9.40 ± 0.09^{c}	2 .00±0.00	0.08± 0.08	9.00± ± 0.06^{e}	1.00 ± 0.00	0.05 ± 0.06
Middle Gangetic Plain Region	9.65 ± 0.11^{a}	12.00 ± 1.00	0.27 ± 0.04	9.46 ± 0.12^{a}	3.00± 0.00	0.12 ± 0.06	9.37 ± 0.31^{c}	6 .00± 1.00	0.18 ± 0.74	9.26±± 0.09^{c}	3.00 ± 1.00	0.12 ± 0.34
Trans-Ganga Plains Region	9.62 ± 0.05^{b}	10.00 ± 2.00	0.24 ± 0.44	9.38 ± 0.54^{b}	2.00 ±1.00	0.08 ± 0.08	9.51 ± 0.09^{a}	4.00 ± 0.00	0.14± 0.19	9.32± ± 0.79^{b}	3 .00± 1.00	0.12 ± 0.51
Southern Plateau and Hills region	9.64 ± 0.08^{a}	9.00 ± 0.00	0.23± 0.12	9.45 ± 0.34^{a}	2.00 ±0.00	0.08± 0.24	9.48 ± 0.45^{b}	5 .00± 1.00	0.16 ± 0.04	9.37± ± 0.91^{a}	2.00 ± 1.00	0.08± 0.07
Total			1.17 ± 0.27			0.41 ± 0.24			0.71 ± 0.52			0.43 ± 0.39

*Values given in column are the average of three replications followed by standard deviation. Values with different alphabetical (a–e) superscripts within a column are significantly different as determined by LSD test (α=0.05).
